# Inflammatory extracellular vesicles prompt heart dysfunction via TRL4-dependent NF-κB activation

**DOI:** 10.7150/thno.39072

**Published:** 2020-02-03

**Authors:** Vanessa Biemmi, Giuseppina Milano, Alessandra Ciullo, Elisabetta Cervio, Jacopo Burrello, Michele Dei Cas, Rita Paroni, Tiziano Tallone, Tiziano Moccetti, Giovanni Pedrazzini, Sarah Longnus, Giuseppe Vassalli, Lucio Barile

**Affiliations:** 1 Laboratory for Cardiovascular Theranostics, Cardiocentro Ticino Foundation, Lugano, Switzerland; 2 Laboratory of Cellular and Molecular Cardiology, Cardiocentro Ticino Foundation, Lugano, Switzerland; 3 Dept. Cœur-Vaisseaux, Centre Hospitalier Universitaire Vaudois, Lausanne, Switzerland; 4 Department of Health Sciences of the University of Milan, Milan, Italy; 5 Cell and Biomedical Technologies Unit Cardiocentro Ticino Foundation, Lugano, Switzerland; 6 Department of Cardiology, Cardiocentro Ticino Foundation, Lugano, Switzerland; 7 Department of Cardiovascular Surgery, Inselspital, Bern University Hospital, Bern, Switzerland; 8 Faculty of Biomedical Sciences, Università della Svizzera Italiana, Lugano, Switzerland

**Keywords:** myocardial infarction, inflammatory extracellular vesicles, macrophages, TLR4 axis

## Abstract

**Background**: After myocardial infarction, necrotic cardiomyocytes release damage-associated proteins that stimulate innate immune pathways and macrophage tissue infiltration, which drives inflammation and myocardial remodeling. Circulating inflammatory extracellular vesicles play a crucial role in the acute and chronic phases of ischemia, in terms of inflammatory progression. In this study, we hypothesize that the paracrine effect mediated by these vesicles induces direct cytotoxicity in cardiomyocytes. Thus, we examined whether reducing the generation of inflammatory vesicles within the first few hours after the ischemic event ameliorates cardiac outcome at short and long time points.

**Methods**: Myocardial infarction was induced in rats that were previously injected intraperitoneally with a chemical inhibitor of extracellular-vesicle biogenesis. Heart global function was assessed by echocardiography performed at 7, 14 and 28 days after MI. Cardiac outcome was also evaluated by hemodynamic analysis at sacrifice. Cytotoxic effects of circulating EV were evaluated *ex-vivo* in a Langendorff, system by measuring the level of cardiac troponin I (cTnI) in the perfusate. Mechanisms undergoing cytotoxic effects of EV derived from pro-inflammatory macrophages (M1) were studied *in-vitro* in primary rat neonatal cardiomyocytes.

**Results**: Inflammatory response following myocardial infarction dramatically increased the number of circulating extracellular vesicles carrying alarmins such as IL-1α, IL-1β and Rantes. Reducing the boost in inflammatory vesicles during the acute phase of ischemia resulted in preserved left ventricular ejection fraction *in vivo*. Hemodynamic analysis confirmed functional recovery by displaying higher velocity of left ventricular relaxation and improved contractility. When added to the perfusate of isolated hearts, post-infarction circulating vesicles induced significantly more cell death in adult cardiomyocytes, as assessed by cTnI release, comparing to circulating vesicles isolated from healthy (non-infarcted) rats. *In vitro* inflammatory extracellular vesicles induce cell death by driving nuclear translocation of NF-κB into nuclei of cardiomyocytes.

**Conclusion**: Our data suggest that targeting circulating extracellular vesicles during the acute phase of myocardial infarction may offer an effective therapeutic approach to preserve function of ischemic heart.

## Introduction

Ischemic injury during the acute phase of myocardial infarction (MI) leads to initiation of a prompt inflammatory response. Cellular debris and metabolic products such as ATP, ions and adenosine, act as damage-associated molecular patterns (DAMPs), inducing the so-called “sterile inflammation” as it is not associated with pathogen 1, 2. DAMPs bind specific pattern recognition receptors (PRRs) on the surface of surviving cells surrounding the necrotic lesion, including adult cardiomyocytes (CM), thus activating macromolecules to aggregate and form inflammasome 1, 3. Nucleotide-binding oligomerization domain-like receptors (NLRs) are tripartite large proteins involved in formation of the inflammasome, recruitment of pro-caspase-1, and promotion of autocatalytic activation of the pro-inflammatory cytokines IL-1β, IL-18 as well as the release of IL-1α, HMGB1 and other chemokines 1, 4. Released cytokines recruit leukocytes (neutrophils, monocytes/ macrophages) from the bone marrow and spleen to the site of injury 5, 6. Infiltrating macrophages (MΦ), once homed into the necrotic area, undergo biphasic activation: MΦ exhibit a pro-inflammatory M1 phenotype early and become polarized toward an anti-inflammatory M2 phenotype later post- MI 7. The systemic release of different subsets of cytokines and proteins by polarized M1/M2 macrophages implies the activation of toll-like receptor (TRLs) and subsequent NF-κB signalling cascades 8. This paracrine secretion may be responsible for accelerated myocardial cell death during the acute phase of MI 9, 10.

In addition to the conventional ER-Golgi secretory pathway, proteins and alarmins are segregated in membrane-enclosed compartments and secreted in nanosized extracellular vesicles (EV) 11, 12. Importantly, inflammosome activity correlates with enhanced secretion of EV and modulation of their cargo 11, 13. Furthermore, both the amount and content of plasma-derived EV correlate with severity of cardiac injury 14. In patients the acute phase of MI, transiently increases concentrations of platelet-, endothelial cell-, and leucocyte-derived microparticles and EV 15-17. In this context it is important being aware that EV cargo (proteins, mRNA, microRNA etc.) not only represents a measurable indicator of biological state or condition, it serves to deliver bioactive molecules from cells of origin to recipient cells and contribute to processes, such as atherosclerosis, thrombosis, and inflammation 18-20. EV smallest fraction, are at least in part responsible for cardiac dysfunction in a model of LPS-induced sepsis 21. In this model, LPS treatment enhances the release of exosomes by macrophages that act as autocrine and paracrine signal mediators and contribute to impair cardiac function 21. While it is well accepted that circulating EV are implicated in exacerbating inflammatory responses after MI, their direct effects on CM remain largely uncharacterized.

We hypothesized that circulating inflammatory EV might have direct cytotoxic effects on CM. We used an *ex vivo* Langendorff system to directly assess whether post-MI plasma-derived EV induce cell death in CM, avoiding interference by other systemic effects. *In vivo*, we sought to evaluate whether the blockade of EV release concomitant with the acute ischemic event reduces myocardial injury, attenuates adverse cardiac remodeling and ameliorates heart function in both the short- and long- term after MI. Finally we explored the *in vitro* mechanisms underlying the effect of M1- and M2-derived EV on CM.

## Results

### Post-infarction circulating extracellular vesicles exert direct cytotoxic effects on cardiomyocytes

**Figure 1:**
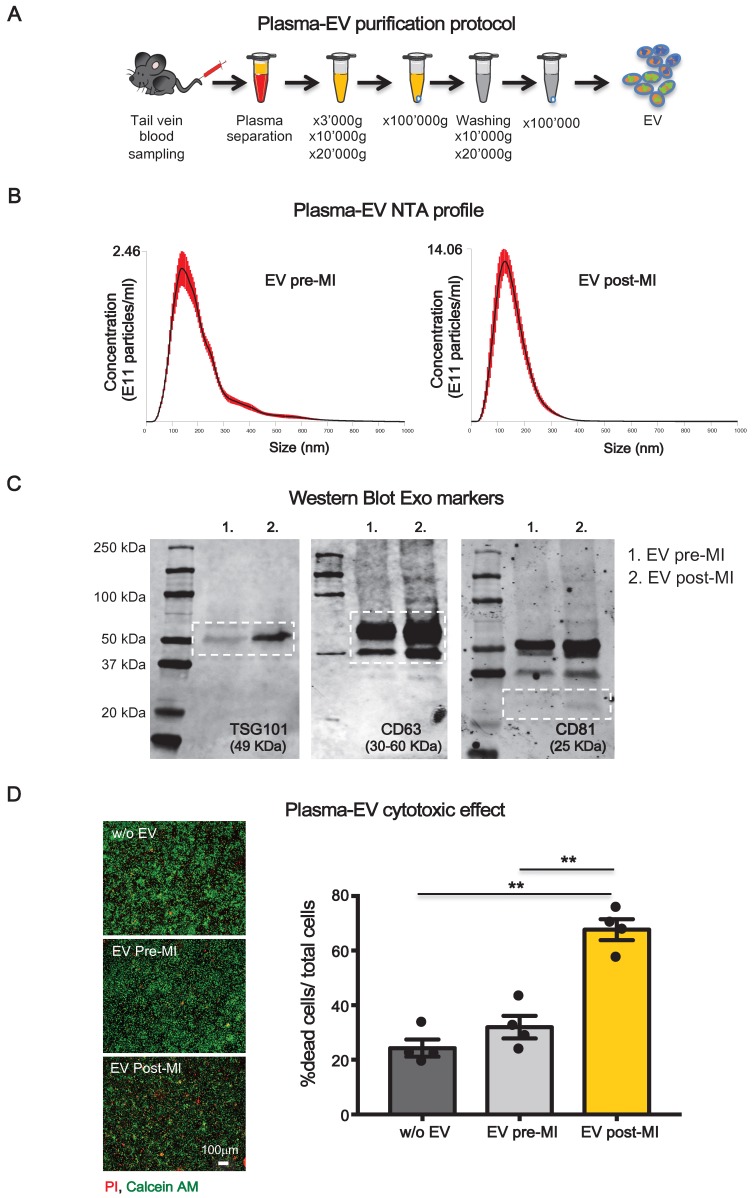
** Plasma-derived EV characterizzation.** (**A**) Plasma derived EV purification protocol. (**B**) Dynamic light scatter analyses of particle size and concentration of plasma derived EV before (pre-MI) and after (post-MI) myocardial infarction (n=5 repeated measurements of 5 different plasma samples per group), red lines represent standard deviations. (**C**) Western blot analysis of specific exosomal markers TSG101, CD63 and CD81. (**D**) Quantification of plasma derived EV cytotoxicity on rat primary neonatal cardiomyocytes. n=4 independent experiments treated with 4 different pools of EV. Left panels are representative images of viability assay for the conditions without EV (w/o EV), EV derived from plasma before (EV pre-MI) and after (EV post-MI) myocardial infarction.Viable cells stain green, dead cells red. All data are presented as mean ± SEM and analyzed by one-way analyses of variance-ANOVA with post-hoc multiple comparisons using the Bonferroni correction (**p < 0.01). Mean, SEM and statistics are reported in full in Table S1.

EV were isolated from blood samples of rats before and 24 hrs after coronary ligation. EV were isolated using serial centrifugation procedure followed by washing step through the resuspension of the pellet and repeating the centrifugation steps as depicted in Figure 1A (see methods). This protocol allows us to purify plasma-derived EV that were enriched in exosomal fraction as indicated by the expression of typical markers of endosomal-derived vesicles such as TSG101, CD63 (Figure S1A) and confirmed by transmission electron microscopy analysis (TEM) (Figure S1B).The absence of contaminants was verified by immunoblot for plasma specific proteins such as apolipoprotein A1 and albumin (Figure S1A) 22. Moreover, a large subpopulation of particles showed a size consistent with exosomes (50-150 nm) as assessed by Nanoparticle Tracking Analysis (NTA) (Figure S1C). However, because EV preparations were not homogeneous, we used the term EV, which is inclusive, but not restricted to exosomes throughout the manuscript.

Although size distribution of isolated EV did not differ pre- and post-MI, the number of circulating EV was significantly increased 24 hrs after MI (EV post-MI) compared to before MI (EV pre-MI) as assessed by NTA (Figure 1B). This trend was also appreciable by WB for the expression of EV markers TSG101, CD63 and CD81 (Figure 1C).

To test whether plasma-derived circulating EV may behave differently on CM when isolated before or after MI, primary neonatal rat myocytes (NRVM) were exposed to 10^7^particles/cm^2^ for 12 hrs in a serum free condition. EV post-MI, but not pre-MI, induced cell death in NRVM (Figure 1D).

### GW4869 reduces the number of circulating extracellular vesicles and modifies their pro-inflammatory cytokine cargo

**Figure 2:**
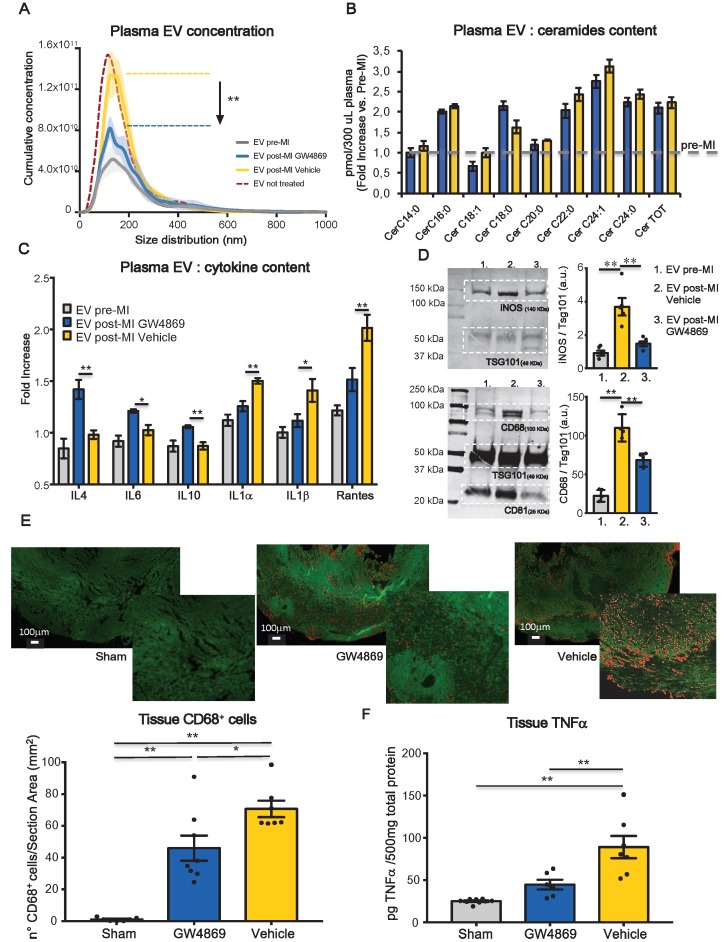
** Systemic inhibition of extracellular vesicles release regulates inflammation in heart after MI.** (**A**) NTA analysis, cumulative curves for concentration and size distribution of plasma-derived EV. EV pre-MI (grey; n=5 repeated measurements of 14 different plasma samples), EV post MI GW4869 (blue; n=5 repeated measurements of 7 different plasma samples), EV post MI vehicle (yellow; n=5 repeated measurements of 7 different plasma samples) and EV post MI from not injected animals (reference line bordeaux). (**B**) LC-MS/MS quantification of EV ceramide content for EV pre-MI (gray reference line), EV post-MI vehicle (yallow bars) and EV post-MI GW4869 (blue bars), n=6 different plasma samples. All data are presented as mean ± SEM pmol/300ml plasma samples and normalized over pre-MI ceramide levels (fold increase vs EV pre-MI). (**C**) EV cytokine content analyses. Data are presented as mean fold change (over blank) ± SEM of 4 independent experiments. (**D**) Western blotting analysis and relative quantification of specific exosomal markers TSG101, CD63 and CD81 and specific inflammatory macrophages markers iNOS and CD68. (**E**) Representative images and quantification of infiltrated CD68^+^ (stained in red) macrophages in heart section 24 hrs post-MI (Sham: n=7, GW4869: n=8, vehicle: n=7 heart section/group). (**F**) Quantification of TNFa in heart tissue 24hrs post-MI (Sham: n=9, GW4869: n=6, vehicle: n=7 heart section/group). All data are presented as mean ± SEM and analyzed by one-way ANOVAs with post-hoc multiple comparisons using the Bonferroni correction (*p < 0.05, **p < 0.01). Mean, SEM and statistics are reported in full in Table S2.

We sought to determine if the blockade of EV release during the acute phase of MI would diminish adverse cardiac remodelling and ameliorate heart function. We used GW4869 as chemical inhibitor as it has been shown to block the secretion of EV *in vitro* 23, 24 and *in vivo* after IP injection 21, 25, 26. One hour before coronary ligation, rats were injected IP with either GW4869 (5mg/kg) or saline solution added of DMSO which was used to dissolve the compound (Vehicle). NTA analysis (Figure 2A) showed that MI significantly increased the total number of circulating EV in rat plasma at 24 hrs compared to baseline (pre-MI). The increased number of circulating EV post-MI, was not affected by the presence of DMSO in vehicle solution as the concentration of EV per ml was comparable with that of animals undergoing MI but not subjected to IP injection (Figure 2A). The boost of EV was reduced by approximately 50-60% in animals pre-treated with GW4869 (Figure 2A). Interestingly the number of total circulating EV drops down at basal level at 48hrs post-MI in both, control and treated animals. Thus, a single injection of GW4869, before the induction of MI, only affect the release of EV in time window of approximately 24 hrs (Figure S2A).

GW4869 inhibits neutral SMase (nSMases) and reduces the secretion of EV by blocking the ceramide-dependent budding of intraluminal vesicles (ILV) into the lumen of multivesicular bodies (MVB) 27. Since it is well known that ceramide is a bioactive sphingolipid involved in many biological functions such as proliferation, apoptosis, differentiation and inflammation,^28^ we assessed by LC-MS/MS whether the ceramide composition of circulating EV was impaired by the GW4869 treatment. The large majority of ceramide isoforms measured in EV samples isolated from 300 μl of plasma, was increased after MI as compared to baseline. The treatment with GW4869 does not affect ceramide isoforms composition as compared to vehicle injected animals (Figure 2B). We also assessed the composition of EV content in terms of inflammatory markers and cytokines. 24 hrs after MI, plasma-derived EV were enriched with pro-inflammatory cytokines IL1α, IL1β and Rantes. EV of animals receiving GW4869 enclosed M2-associated cytokines such as IL4, IL6 and IL10 (Figure 2C). As shown in a previuos papers, EV not only transfer their protein to the target cells, they are also able to transfer mRNA 29, 30. Thus, we verified whether the cargo of pro-inflammatory cytokines that was observed following MI, was also accompanied by the presence of specific mRNA encoding for those cytokines. As showed in supplementary figure 2B, EV cargo does not contain mRNA for iNOS, INFγ, IL1α. The presence of vesicle mRNA was detected for IL1β, Rantes, IL6. However, the level of expression did not change between groups (Figure S2B). EV post-MI Vehicle expressed higher levels of inflammatory markers iNOS and CD68, as compared to EV pre-MI and EV post-MI GW4869 (Figure 2D). Moreover, in GW4869 pre-treated animals resulted in a diminished cell infiltration (CD68^+^ cells) in the heart tissue as well as reduced TNF-α (Figure 2E-F), thereby confirming that circulating EV may exacerbate inflammatory responses during the acute phase of MI.

### Inhibition of extracellular vesicles release mitigate myocardial dysfunction in rat model of myocardial infarction

**Figure 3:**
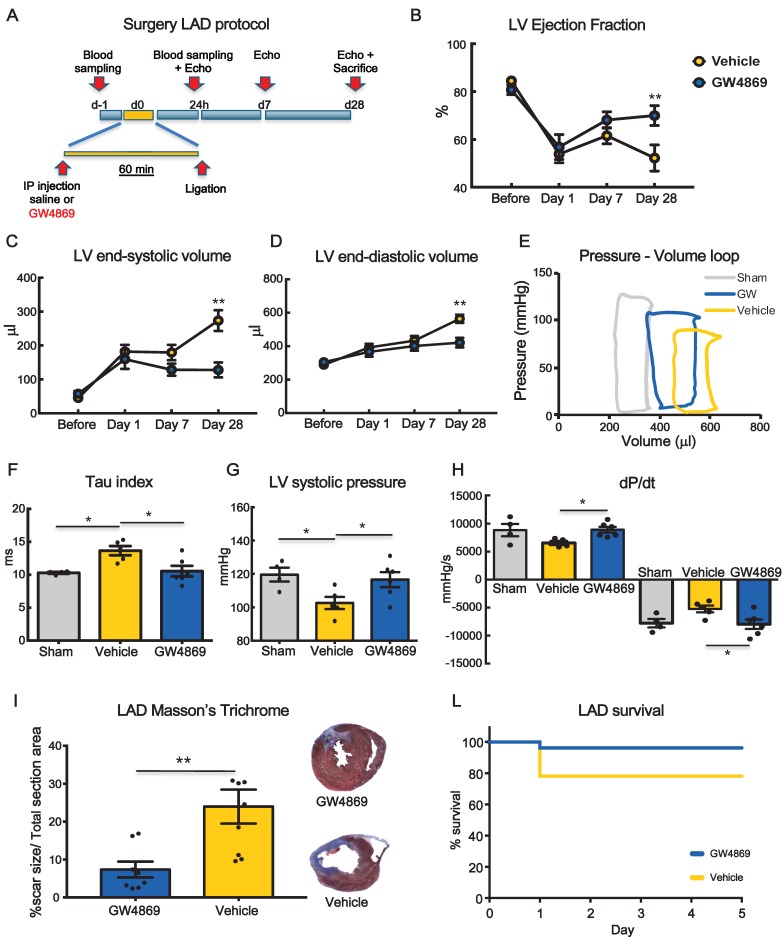
** Systemic inhibition of extracellular vesicle release mitigates myocardial dysfunction after permanent coronary artery ligation.** (**A**) Scheme depicting the study protocol. LAD occlusion was induced in rats one hour after IP injection of GW4869 or vehicle. (**B-D**) Echocardiography evaluation of LVEF, LVSV and LVDV (n= 6 rats/group). Data ara analyzed using two-way ANOVAs followed by Bonferroni post-hoc test. Data are presented as mean ± SEM (*p < 0.05, **p < 0.01) (**E**) Representative LV pressure-volume cardiac loops. (**F-H**) Hemodynamic analyses of LV systolic pressure, LV relaxation velocity (tau index) and LV contractility capacity (dP/dt) (n=4 sham, n=5 vehicle, n=6 GW4869 treated animals). Data are presented as mean ± SEM. *p < 0.05, **p < 0.01 (one-way ANOVA with post-hoc multiple comparisons using Bonferroni multiple comparisons correction). (**I**) Masson's trichrome staining for quantification of infarct size (n = 8 animals per group). The percentage of scar size was calculated by normalizing blue fibrotic area by total section area. Variables are presented as mean ± SEM and analyzed by Student's T test **p < 0.01. (**J**) Kaplan-Meier survival curve. (GW4869 survival= 96.15% n=26 animals, Vehicle survival = 78.12% n= 32 animals; HR 4.44; 95%-CI 1.01-19.65; p = 0.04). Mean, SEM and statistics are reported in full in Table S3.

To monitor the functional effects of GW4869, rats underwent echocardiography at 1 day (baseline), 7 and 28 days after MI (Figure 3A). Left ventricular ejection fraction (LVEF) was significantly decreased at 24 hrs after MI in both groups. At 7 days, there was a trend in preservation of LVEF in GW4869 injected animals as compared to vehicle which became statistically significant at 28 days (Figure 3B). Parallel changes in left ventricle (LV) end-systolic and LV end-diastolic volumes were observed (Figure 3C-D). To confirm echo analysis a subset of animals were subjected to hemodynamic measurements by Millar catheter. From LV pressure/volume loops (Figure 3E), the time constant of isovolumic LV pressure (Tau) was significantly increased in vehicle injected rats and rescued to control levels in GW4869 injected rats, indicating improved diastolic relaxation (Figure 3F). Moreover, GW4869 significantly increased LV systolic pressure (Figure 3G) and improved dP/dt max and -dP/dt min (Figure 3H). Histological analysis displayed smaller scar in GW4869 group as compared to vehicle (Figure 3I). Pre-treatment of rats with GW4869 significantly improved animal survival after MI compared to vehicle pre-treated animals (Figure 3J).

**Figure 4:**
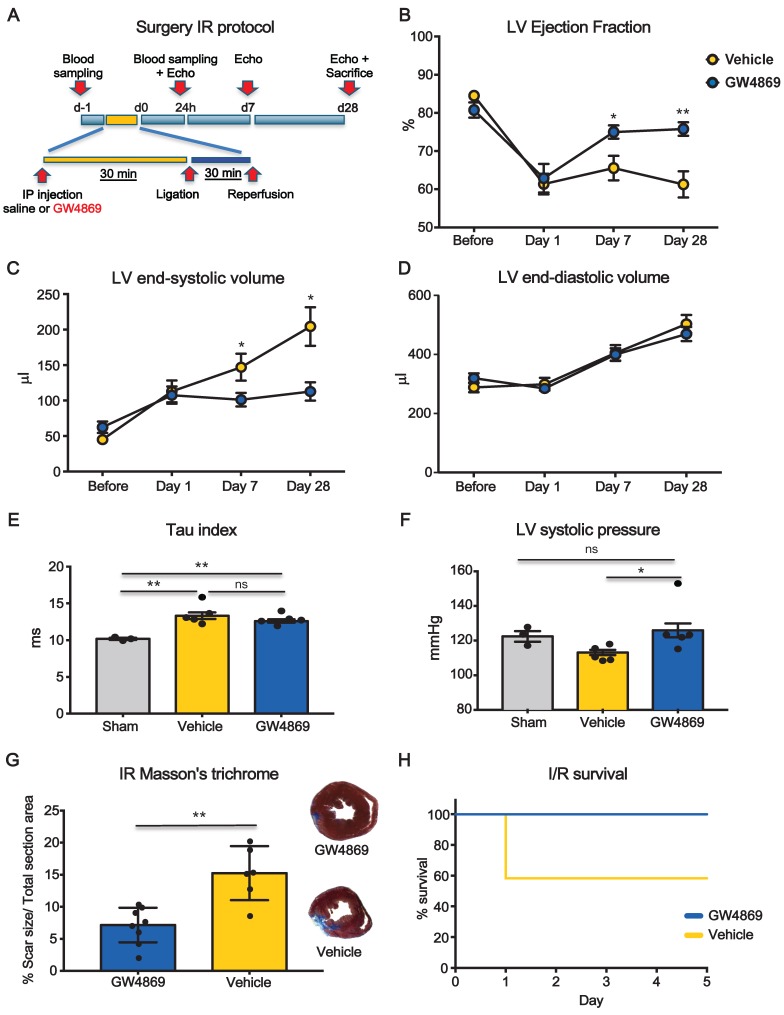
** Systemic inhibition of extracellular vesicle release mitigates myocardial dysfunction after ischemia-reperfusion injury.** (**A**) Scheme depicting the study protocol. I/R model was induced by ligating the left descending anterior coronary that was then released after 30 min. Rats underwent IP injection of GW4869 or vehicle one hour before coronary reperfusion. (**B-D**) Echocardiographic evaluation of LVEF, LVSV and LVDV (n=6 rats/group). Data were analyzed using two-way ANOVAs followed by Bonferroni post-hoc tests. Data are presented as mean ± SEM (*p < 0.05, **p < 0.01). (**E-F**) Hemodynamic analysis of LV relaxation velocity (tau index) and LV systolic pressure (n=3 sham, n=5 vehicle, n=5 GW4869 treated animals). Data are presented as mean ± SEM. *p < 0.05, **p < 0.01 (one-way ANOVAs with post-hoc Bonferroni multiple comparisons correction). (**G**) Masson's trichrome staining for quantification of infarct size (n =8 GW4869 rats, n=6 vehicle animals). The percentage of scar size was calculated by normalizing blue fibrotic area with total section area. Variables are presented as mean ± SEM and analyzed by Student's T test **p < 0.01. (**H**) Kaplan-Meier survival curve (GW4869 survival= 100% n=10 animals, Vehicle survival = 58.33 % n= 12 animals; HR 9.63; 95%-CI 1.36-68.12; p = 0.023). Mean, SEM and statistics are reported in full in Table S4.

We performed additional set of experiments to test whether the inhibition of EV release was also protective in the context of ischemia-reperfusion (I/R) which is generally accepted as more translational model compared to permanent coronary artery ligation (LAD) (Figure 4A). LVEF was significantly decreased at day 1 after I/R in both groups. At 7 days LVEF was significant higher in GW4869 injected animals as compared to vehicle and it was preserved at 28 days (Figure 4B). Parallel changes in LV end-systolic volumes were observed, whereas there was no difference in LV end-diastolic volumes (Figure 4C-D). Although the LV Tau constant was not significantly different between vehicle and GW4869 (Figure 4E) the latter showed significantly increased LV systolic pressure (Figure 4F). In animals pre-treated with GW4869, scar size, as assessed by Masson's trichrome staining, was significantly reduced as was mortality rate (Figure 4G-H).

### *Ex vivo* assessment of effects of extracellular vesicles on cardiomyocyte cytotoxicity

**Figure 5:**
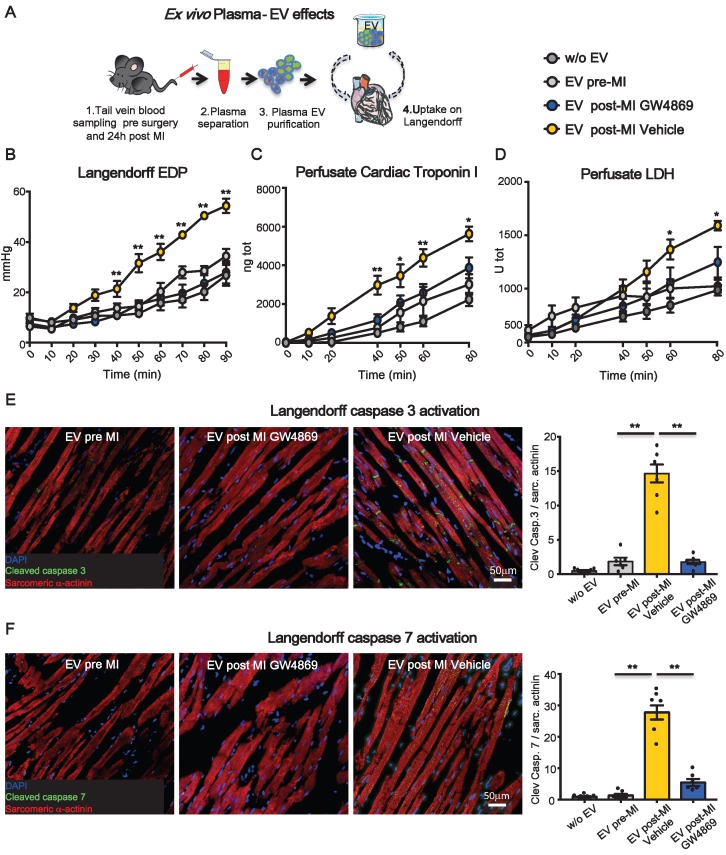
***Ex-vivo* cytotoxic effects of plasma-derived extracellular vesicles on cardiomyocytes***.* **(A)** Experimental design. Normal rat hearts were retrogradely perfused for 90 min. PBS, EV pre-MI, EV post-MI GW4869 or EV post-MI Vehicle were added to the perfusion solution. **(B)** Evaluation of end-diastolic pressure (w/o EV: n=5; EV pre-MI: n=5; EV post-MI GW4869: n=6; EV post-MI Vehicle: n=6). **(C)** Cardiac troponin I levels in Langendorff perfusate (w/o EV: n=5; EV pre-MI: n=5; EV post-MI GW4869: n=6; EV Post-MI Vehicle: n=6). **(D)** LDH levels in Langendorff perfusate (w/o EV: n=5; EV pre-MI: n=5; EV post-MI GW4869: n=6; EV post-MI Vehicle: n=6) **(E-F)**. Representative images of immunofluoresce for the cleaved caspase 3 and cleaved caspase 7 in heart tissue after 2 hrs of perfusion with plasma derived EV (w/o EV: n=6; EV pre-MI: n=6; EV post-MI GW4869: n=6; EV post-MI Vehicle: n=6). Cleaved caspeses stain green, cardiomyocytes stain red. Data are analyzed using two-way ANOVAs followed by Bonferroni post-hoc test and are presented as mean ± SEM. **p<0.01 EV post-MI Vehicle vs. either EV post-MI GW4869, EV pre-MI, and w/o EV. Mean, SEM and statistics are reported in full in Table S5.

To assess whether the impairment of heart function was due to direct cytotoxic effects of circulating EV on adult CM and to avoid interference of circulating cells as source of soluble factors and vesicles, an acellular physiologic solution containing plasma-derived EV was used to perfuse normal hearts in a retrograde (Langendorff) *ex vivo* system. Equal amounts (5 x 10^10^) of circulating EV pre-MI and post-MI from vehicle (EV post-MI Vehicle) or GW4869 (EV post-MI GW4869) injected animals, were added to the perfusate and circulated for 2 hrs (Figure 5A). Saline without EV was used as control solution. Heart function was evaluated by end diastolic pressure (EDP) and CM death was assessed by measuring the release of cTnI and LDH into perfusate. EV-Vehicle was detrimental on heart function as early as 40 minutes. Both EDP and cTnI level increased over time with the physiological worsening of heart function occurring in the presence of control solution (Figure 5B-C). Although at later time point respect to the increasing level of cTnI, the release of LDH was also augmented in hearts perfused with EV post-MI Vehicle (Figure 5D). Moreover, tissue immunofluorescence of *ex vivo* perfused heart revealed a striking expression of cleaved caspase 3 and 7 in EV post-MI Vehicle group as compared to pre-MI and EV post-MI GW4869 (Figure 5E-F). Data on activation of caspases were also confirmed *in vitro* in NRVM (Figure S3). All together these data showed that independently from necrotic massive event subsequent to acute ischemia, circulating EV act directly on viable CM, inducing cell death via mechanisms retrieving apoptosis and pyroptosis.

### Plasma-derived extracellular vesicles from a rat model of ischemia induce cytotoxic effects in cardiomyocytes through activation of the TLR4/NF-κB axis

**Figure 6:**
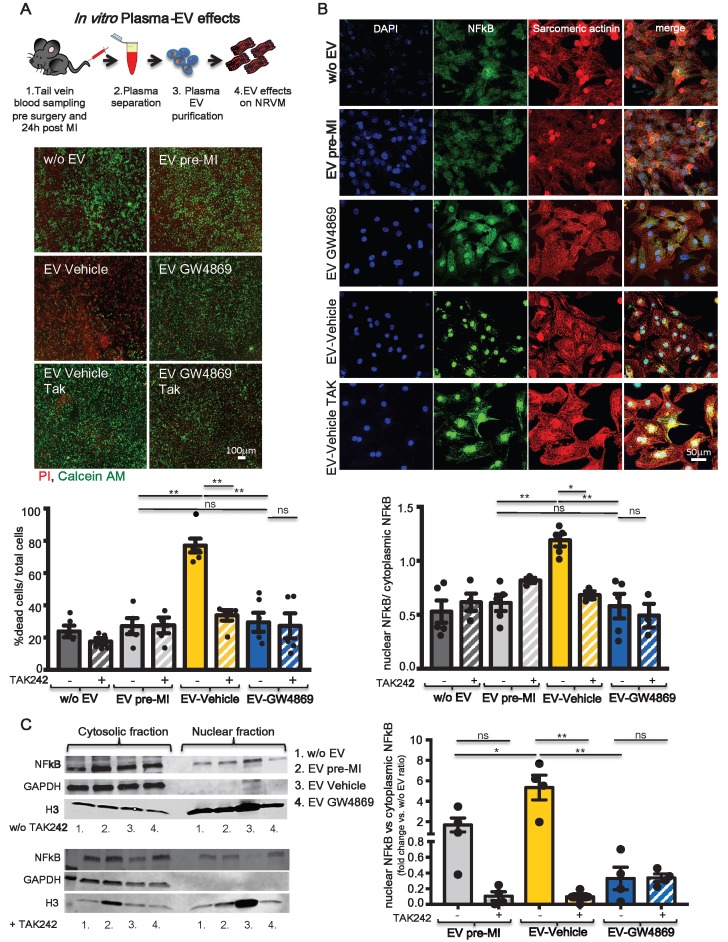
** TLR4-NFkB axis regulates *in vitro* plasma-derived EV effects. (A)** Quantification of plasma derived EV cytotoxicity on rat primary neonatal cardiomyocytes (NRVM) using the TLR4-specific inhibitor TAK242. Representative images of 5 independent experiments treated with 5 different pools of EV. Viable cells stain green, dead cells red. **(B)** Evaluation of NFkB nuclear translocation in NRVM. DAPI mask was used to detect NFkB nuclear fraction. Dots in graphs represent the number of different plasma-derived EV tested in each experimental condition. **(C)** WB analysis and quantification of nuclear translocation of NFkB (n=4 independent experiments). Quantification data are presented as nuclear/cytosol ratio within the same treatment and expressed as mean fluorescent intensity fold change versus w/o EV. All data are presented as mean ± SEM and analyzed by one-way ANOVAs with post-hoc Bonferroni multiple comparisons correction (*p < 0.05, **p < 0.01). Mean, SEM and statistics are reported in full in Table S6.

A recent paper by Dalvi et.al showed that EV derived from immune-activated monocytes stimulate TLR4/MyD88 pathway thereby activating NF-κB 31. Cardiomyocytes express the TLR4 receptor 32 and its expression and activation in infarcted and remote areas following MI contributes to LV remodelling and functional impairment 33. Inhibition of TLR4 has been described as anti-apoptotic in an animal model of ischemia/reperfusion 34. Therefore we checked *in vitro* whether pro-inflammatory circulating EV act on NRVM through this axis. 1x10^7^ EV/cm^2^ derived from plasma of pre-MI rats and vehicle or GW4869 treated and infarcted animals were added to the medium of NRVM. As expected, EV-Vehicle induced significantly higher cell death in NRVM as compared to EV-GW4869. The latter was not different from control treatments (EV pre-MI and medium w/o EV). The cytotoxic effect of EV-Vehicle was abolished when NRVM were pre-treated with the TLR4 specific inhibitor TAK242 (Figure 6A). We also verified whether the stimulation of TLR4 receptor induced the transloncation of NF-κB into nuclei of NRVM. Both immunofluorescence assay (Figure 6B) and Western Blots on cytosolic and nuclear fractions (Figure 6C), confirmed this hypothesis. The activation and translocation of NF-κB was inhibited by TAK242. Nuclear translocation of NF-κB was also assesed *ex vivo*, in adult CM isolated from healty heart perfused with EV-Vehicle for 2 hours (Figure S4). These data confirmed that the TLR4/NFκB axis plays an important role in mediating EV-induced cell death on CM.

### Extracellular vesicles from pro-inflammatory bone marrow-derived macrophages are cytotoxic *in vitro*

**Figure 7:**
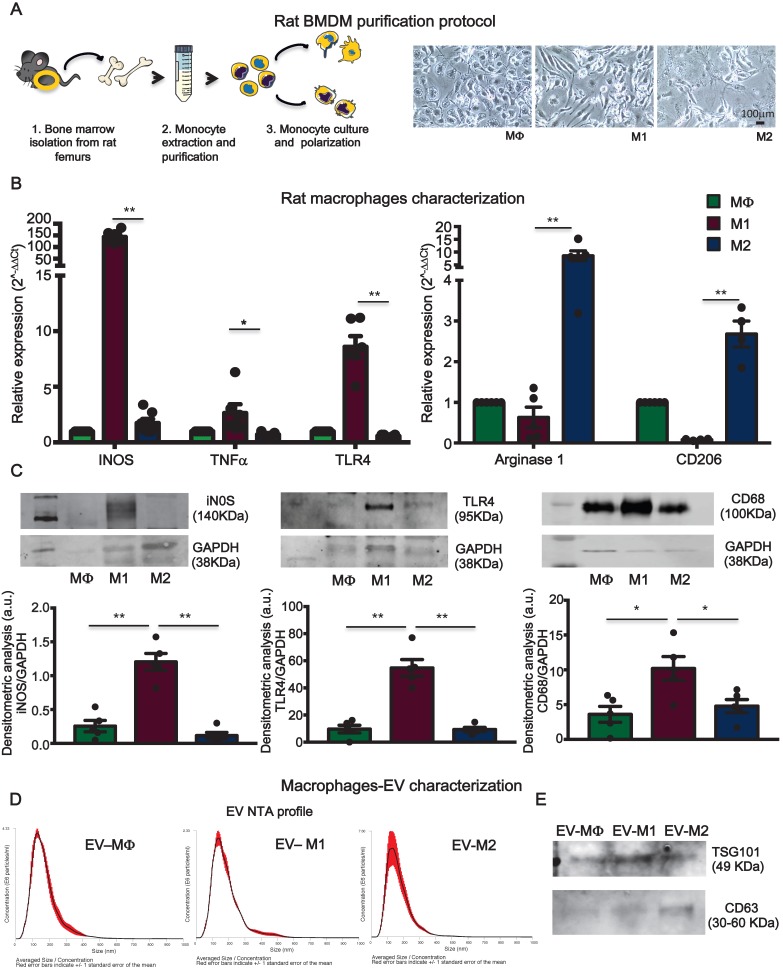
** Bone marrow-derived macrophages and extracellular vesicles characterization***.*** (A)** Experimental design of bone marrow-derived macrophage purification and polarization protocol. **(B)** Relative expression of specific M1 and M2 genes**.** Data show 2^-DDCt^ values vs. MF macrophages; n=6 independent experiments (M1 markers); n=4 independent experiments (M2 marker).** (C)** Western blot analysis of specific macrophage markers. Quantitative densitometric data were normalized by GAPDH (n=5 independent experiments).** (D)** Representative dynamic light scatter profile of particle size and concentration of BMDM derived EV. Red lines represent standard deviations. **(E)** Western blot analysis of specific exosomal markers TSG101 and CD63. All data are presented as mean ± SEM and analyzed by one-way ANOVAs with post-hoc multiple comparisons using Bonferroni correction (*p < 0.05, **p < 0.01). Mean, SEM and statistics are reported in full in Table S7.

**Figure 8:**
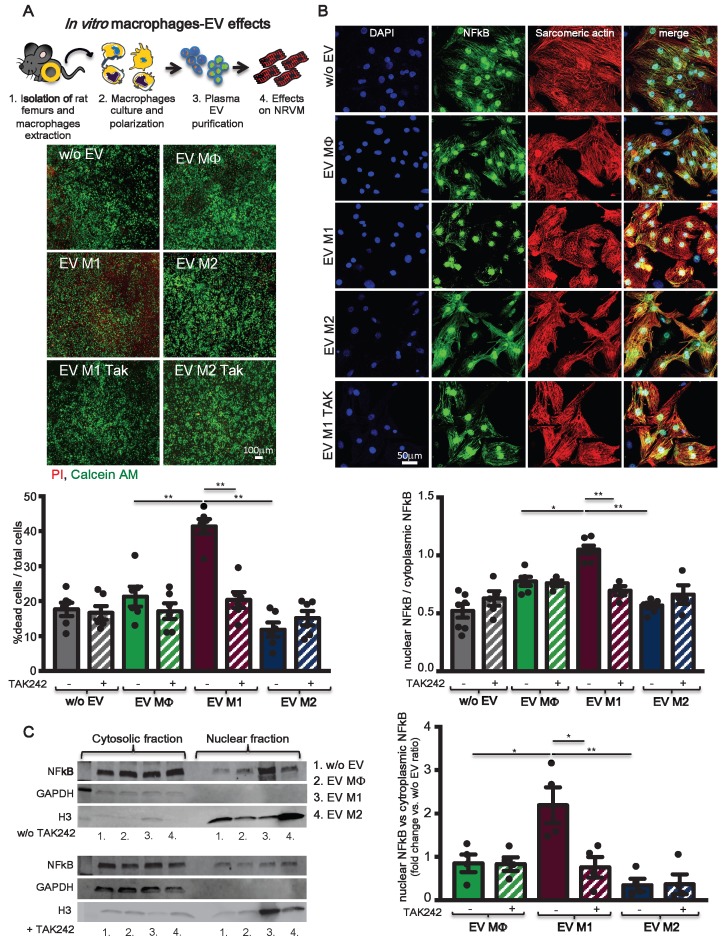
** TLR4-NFkB axis regulates *in vitro* macrophages-derived EV effects. (A)** Evaluation of BMDM derived EV cytotoxicity on rat primary neonatal cardiomyocytes using the TLR4-specific inhibitor TAK242. Data refers to 6 independent experiments treated with 6 different pools of EV. Viable cells stain green, dead cells red. **(B)** Evaluation of NFkB nuclear translocation in NRVM using the TLR4-specific inhibitor TAK242. DAPI mask was used to detect NFkB nuclear fraction. Dots in graphs present the numbers of different BMDM-derived EV tested in each experimental condition. **(C)** WB analysis and quantification of nuclear translocation of NFkB using the TLR4-specific inhibitor TAK242 (n= 4 independent experiments). Quantification data are presented as nuclear/cytosol ratio within the same treatment and expressed as mean fold change in fluorescence intensity versus w/o EV. All data are presented as mean ± SEM and analyzed by one-way ANOVAs with post-hoc multiple comparisons using Bonferroni correction (*p < 0.05, **p < 0.01). Mean, SEM and statistics are reported in full in Table S8.

To assess the hypothesis that circulating pro-inflammatory EV mostly derive from activated macrophages, rat bone marrow-derived primary monocytes were isolated and cultured *in vitro*. Monocytes were first differentiated into macrophages (MΦ) and subsequently polarized to M1 or M2, by supplementing the culture medium with specific cytokines (Figure 7A, see methods). Macrophages were characterized for mRNA expression of specific transcripts. M1 express typical pro-inflammatory markers such as iNOS, TNFα and TLR4 whereas, M2 express Arginase1 and CD206 (Figure 7B). M1 and M2 polarization was also confirmed by WB for the expression of iNOS, TLR4 and increased expression of CD68 as compared to MΦ and M2 (Figure 7C). EV derived from conditioned medium of cultured cells were profiled for NTA analysis (Figure 7D) and characterized for the expression of exosomes markers such as TSG101, CD63 and Alix (Figure 7E; Figure S1B). NTA profiles showed typical size distribution of exosomes or small EV (Figure 7D). Isolated EV were used in a functional assay with NRVM. EV released by M1, but not those released from M2, induced cell death in normoxic NRVM (Figure 8A). To evaluate the effects of macrophages-derived EV on CM subjected to ischemia, we performed functional assay in condition of FBS starvation at 1% of O_2._ As aspected, the rate of cells death was augmented in all conditions as compared to normoxia ( Figure 8A, Figure S5). In presence of M1-derived EV, CM mortality reached 80% (Figure S5) which is double as compared to normoxia (Figure 8A). Moreover, EV derived from medium conditioned by M1 activated nuclear translocation of NF-κB via TLR4 (Figure 8B-C). Specific inhibition by TAK242 of TLR4 receptor activity abrogated NF-κB translocation and reduced cytotoxic effects. Finally to asses whether protein present onto surface of EV are important for the signaling cascade leading to cytotoxic effect, we performed acid-washing procedure (see method) aiming to remove surface antigens by incubating vesicles for 5 min with acid glycine 35. The efficency of protocol was monitored by dot-plots immunoassay against CD63 known to be abundantly expressed on the surface of EV (Figure S6A). Acid-washed EV Vehicle and EV M1 had reduced toxicity as compared to untreated counterpart EV (Figure S6B-C). These findings suggest that EV surface proteins contribute to EV-mediated cell toxicity on CM.

## Discussion

Numerous preclinical studies have approached ischemic heart disease by targeting different players and cofactors involved in inflammation, occurring during the acute event of MI, to reduce organ remodelling and dysfunction in the heart 36-39. A recombinant human IL-1 receptor antagonist has been tested in a model of ischemia in mice by using immediate or delayed administration (24 hours after ischemia). IL-1 blockade was associated with a significant reduction in cardiomyocyte apoptosis and attenuation of adverse cardiac remodelling in both immediate- and delayed- treatment groups 36. Of note: treatment was administered during surgery (or starting on day 2 in delayed treatment) and then daily for a total of 6 doses. Other studies focused on low-molecular-weight inhibitors to block signalling molecules downstream of TLRs receptors such as NLRP3 40. Similar effects have been observed by inhibiting the purinergic P2X7 receptor, which is activated by ATP released from dying cells 41. Alpha-1 antitrypsin (AAT) a naturally occurring anti-inflammatory protein, has been used in an experimental AMI model in mice and significantly reduced myocardial injury 42. These various strategies have consistently shown that targeting the inflammatory response following MI ameliorates heart function outcome. Taking a similar approach, we focused on circulating EV following ischemic heart injury, as they have been demonstrated to act as crucial mediators of inflammation 43. In patients with ST-segment elevation MI (STEMI), the increased number of EV has been associated with augmented release of particles by platelets, red blood cells, endothelial cells, or leucocytes 44-47. In line with previous studies in mice and humans 48, 49, our data confirms that the number of circulating EV increases during the acute phase of MI. Here we added the findings that these circulating EV have direct cytotoxic effects on CM leading to the worsening of cardiac ischemic damage. In the present study, we assessed this hypothesis by different approaches: *in vitro* by adding EV post-MI into the medium of NRVM*, ex vivo* in isolated, perfused hearts and *in vivo* by inhibiting the release of EV during the very first phase of myocardial infarction. EV derived from plasma of animals that underwent coronary ligation were cytotoxic when added to the medium of NRVM, whereas those derived from plasma of healthy rats did not exert any toxic effects. Post-infarction circulating EV caused CM disruption when added to the perfusate of isolated heart as shown by the increased level of cTnI and LDH (both markers for the MI-diagnosis) and rapidly (within 40 min) worsened heart functionality as assessed by the increase of end diastolic pressure. *In vivo* we used GW4869 as an inhibitor of EV generation and release, as it has been utilized to suppress the secretion of EV from LPS-induced sepsis model 21. EV systemically released during the acute phase of infarct most likely originate from inflammatory cells since their cargo is enriched in IL-1α, IL-1β and Rantes. This was confirmed by the fact that a diminished amount of circulating EV subsequent to the GW4869 treatment was also accompanied by reduction of EV-associated pro-inflammatory cytokines and well-known, pro-inflammatory macrophage markers such as CD68 and iNOS. Moreover, by reducing the number of circulating EV, the inflammatory response at site of injury was attenuated. The number of CD68^+^ infiltrating cells as well as the expression of TNF-α, were significantly reduced in the heart tissue of infarcted rats that received pre-treatment with the inhibitor GW4869 as compared to rats that were infarcted only. Consistently, we observed that intraperitoneal injection of GW4869, one hour before LAD ligation, effectively reduced the burst in circulating EV after MI. A single injection of GW4869 was able to increase the number of surviving CM within 24 hrs, resulting in reduced fibrotic tissue in the long term after MI. Our approach was also validated in an ischemia/reperfusion model as the restoration of blood flow at the earliest possible time point remains the method of choice in all current treatment options of ischemia. We sought to evaluate whether reducing the number of circulating EV, during the acute phase of MI, has still effect on the top of the gold standard clinical practice. Indeed, our data confirmed this concept in I/R model, providing the basis for future investigations, and eventually to explore this approach in a large animal model.

As such, we propose that the detrimental effect directly exploited by inflammatory EV on CM is also accompanied by EV-mediated autocrine signalling which amplifies the quantity of pro-inflammatory cytokines produced by the initial ischemic event and the recruitment of monocytes to the site of injury. This is in line with previous studies showing that EV from body fluids are internalized by the monocytic leukemia THP-1 cell line and activate the release of cytokines including IL-1β in a dose-dependent manner 50.

Plasma-derived EV promote NF-κB nuclear translocation in different cell types 50-52. We demonstrated for the first time that this pathway is also activated in CM upon exposure to inflammatory EV. NF-κB activation is required for EV-mediated cytotoxicity, which is in line with evidence demonstrating that nuclear translocation of NF-κB induces apoptosis in CM 53. We confirmed that EV-mediated triggering of NF-κB activation was strongly impaired by the functional blocking of TLR4 as shown by Bretz et al 50. Collectively, these data implicate that EV have signalling capacity; that they interact with TLR4 on the CM surface, and that this is determinant for signal initiation. As downstream effects of this pathway we showed activation of caspases 3 and 7 in combination with LDH release in healty CM, demonstrating that cell death mechanisms, ascrivable to apoptosis and/or pyroptosis, can contribute to heart function worsening in addition to necrotic events occurring during the acute phase of ischemia. Our data reveal that circulating EV post-MI play a double role in promoting and exacerbating inflammatory response and contributing to reduced heart function by directly inducing CM death. The latter was demonstrated on NRVM *in vitro* but most importantly it was also assessed *ex vivo* on adult CM.

Considering that activated macrophages are a major source of pro-inflammatory cytokines and play a critical role in the pathogenesis of MI, we selected macrophages derived from bone marrow for the *in vitro* model to draw a comparison with the direct, *in-vivo* EV effects on CM. We are aware that resident heart macrophages and EV derived from those cells would have represented a relevant model to study, however it is known that after MI, monocytes recruitment occurs as early as 30 minutes after ischemia and that bone marrow and spleen acts as a reservoir from which monocytes are recruited 9, 54, 55. Heidt et al. suggested that following MI resident cardiac macrophages die and may be replaced by CCR2-expressing monocyte derived cells with potent pro-inflammatory properties 56. Moreover the number of resident heart macrophages is very low in normal condition, thus making their isolation very challenging, even more difficult would be the *in vitro* culture of those cells and the subsequent enrichment of EV into conditioned medium. On the other hand the direct isolation of EV from tissue, remain technically challenging 22. In our experiments EV derived from bone marrow-derived M1 macrophages recapitulated the functional effects of those derived from the plasma of infarcted rats. Although it is generally accepted that activation in M1, but not M2, macrophages contributes to cardiac remodelling after MI 57, a direct causal relationship between the release of EV by M1 and their cytotoxic effect on NRVM has not been demonstrated previously.

Our *in vitro and ex vivo* functional tests were performed by comparing the same number of circulating particles derived from healthy or infarcted rats. Cytotoxic effects might be exacerbated following MI *in vivo* not only by the more noxious nature of the circulating inflammatory EV, but also by the significant increase in their number, thus opening a valid prospective approach for targeting detrimental aspects of the inflammatory process following ischemic injury.

From a translational perspective, the approach used here is limited as the injection of inhibitor was performed before the coronary ligation. To date we do not know whether a single injection post-MI (at time of reperfusion) would provide benefits to a comparable degree. This aspect, as well as the possibility of using more specific approaches (i.e. silencing of proteins implicated in the active transport of intracellular vesicles) to specifically inhibit the release of EV from macrophages in a desirable time window; will be a focus of future studies. As presented in this study we cannot exclude that the beneficial effects of GW4869 on cardiac function is due to multiple effects such as the reduction of exosome release from cells other than macrophages (neutrophils, endothelial cells and platelets) as well as a direct effect on MΦ polarization, that has not been addressed here. The present study provides proof of concept for a new mechanism of EV-induced cytotoxicity, and offers a new perspective for the intervention and treatment of inflammation-related diseases, such as heart acute ischemia.

## Methods

### Animal experiments

Experimental protocols were approved by the Local Veterinary Authority (TI-10-17, TI-08-18). All procedures conformed to the directive 2010/63/EU of the European Parliament. Two different models of myocardial ischemic injury (MI) were used: permanent coronary artery ligation (LAD) and acute ischemia/reperfusion (I/R). MI was induced in healthy Wistar male rats (250-300g body weight) anesthetized with a cocktail of Ketamine (ketasol100, 100mg/Kg) and Xylazine (Rompun 2%, 8mg/Kg), intubated and ventilated. The left anterior descending artery was ligated with a 4.0 silk suture. In I/R operated animals, the coronary ligature was released after 30 min whereas the left coronary artery was not ligated in sham-operated group. The chest was then closed, pneumothorax was reduced and the rats were treated with Meloxicam (0,5mg/kg) for 5 days after surgery. Sixty minutes before coronary artery occlusion, animals were injected intraperitoneally (IP) with a chemical inhibitor of neutral sphingomyelinase (GW4869, Sigma 5mg/Kg) or diluent solution (Vehicle, DMSO 1:3v/v PBS1X). 24 hrs after injection, rats underwent tail vein blood sampling. Sedated rats were subjected to transthoracic echocardiography at 24 hrs, 7 and 28 days post-MI using Vevo2100 echocardiography system (VisualSonic System 2100) equipped with a 15MHz linear transducer. Under ECG monitoring of heart rate, 2D images of hearts were acquired in parasternal long axis and short axis (apex, mid and base levels) views. Left ventricle (LV) ejection fraction (LVEF), LV end-diastolic volume (LVDV) and LV end-systolic volume (LVSV) were measured by Simpson's analysis. Hemodynamic analysis was performed at day 28 using a Millar pressure-volume conductance catheter. Rats were anesthetized with an IP injection of Ketamine (ketasol100, 100mg/Kg) and Xylazine (Rompun 2%, 8mg/Kg). The trachea was cannulated and the animal connected to a rodent ventilator. The catheter was introduced through the right carotid artery into the ascending aorta and then into the LV cavity. After hemodynamic measurements, animals were sacrificed.

### Rat bone marrow-derived macrophages isolation, polarization and characterization

The femur was isolated from euthanized Wistar male rats and marrow was flushed into a cold PBS solution containing 2% fetal bovine serum (FBS). Mechanically dissociated cells were incubated on ice with three volume of 0.8% NH_4_Cl solution to remove red blood cells. 10^6^ cells/cm^2^ were seeded in tissue culture plates (Corning Constar) and cultured in Iscove's Modified Dulbecco's Medium (IMDM, Gibco) supplemented with 10% FBS, 1% v/v penicillin-streptomycin (Life Technologies) 10ng/ml M-CSF (RayBiotech). After 7 days, bone marrow-derived macrophages (MΦ) were polarized to inflammatory M1 with IMDM, 1% v/v penicillin-streptomycin, 10ng/ml M-CSF supplemented with 100ng/ml TNFα (ApexBio) and 100ng/ml IFNγ (RayBiotech). The non-inflammatory BMDM-M2 profile was induced by supplementing the culture medium with 20ng/ml IL-4, 20ng/ml IL-10 and 20ng/ml IL13 (RayBiotech).

### Extracellular vesicles isolation and purification

EV were isolated from citrate plasma samples or macrophages supernatant by differential centrifugation. Briefly, samples were centrifuged at 3000g (20 min) followed by filtration through a 0.2μm membrane and centrifugation at 10000g (30 min) to remove cellular debris. EV were than pelleted by ultracentrifugation at 100000g (18h) using a Beckman Optima Max-TL ultracentrifuge (Beckman Coulter). BMDM-EV pellets were re-suspended in 100µL PBS, pH 7.4, and stored at -80°C. Plasma EV-enriched pellets underwent washing step (by diluition in 3ml PBS 1X ) and further centrifuged at 10000g (20 min) and 20000g (30 min) with a final step of ultracentrifuge at 100000g (18h). Plasma-EV pellets were then re-suspended in 100 µL PBS, pH 7.4, and stored at -80°C. EV numbers and size were measured using Nanosight Technology (Malvern Instruments). EV characterization was assessed by the presence of TSG101 (Abcam #12501, 1:1000), and tetraspanin CD63 (Invitrogen #MA5-31276, 1:1000) CD81 (Invitrogen #MA517937, 1:1000) and the absence of contaminants GRP94 (Abcam #108606, 1:500), Apolipoprotein A1 (Invitrogen #701239, 1:500), Albumin (Abcam, #ab207327, 1:2000).

### Extracellular vesicles cytokine content analysis

EV content of multiple cytokines were simultaneously determined using enzyme-linked immunosorbent assay (Multi-Analyte ELISArray for Rat, Qiagen) following manufacturer's instructions. EV in sample dilution buffer were briefly sonicated and 10^10^ EV/well were analysed. Sample dilution buffer without EV was analysed as experimental blank.

### Lipid extraction and exosomes LC-MS/MS ceramide content quantification

Sphingolipid extraction and LC-MS/MS analysis were performed as already described elsewhere 58. Briefly, sphingolipids from plasma-purified exosomes were extracted using a monophasic chloroform/methanol/water mixture (30:60:10, v/v) and analysed by a LC Dionex 3000 UltiMate (Thermo Fisher Scientific,) coupled to a tandem mass spectrometer AB Sciex 3200 QTRAP (AB Sciex,). Separation was achieved by a reversed-phase BEH C-8 100 x2.1 mm x1.7 µm analytical column by a linear gradient between eluent A (water 0.2% formic acid 2mM ammonium formate) and eluent B (methanol 0.2% formic acid 2mM ammonium formate). Quantitative analysis was performed interpolating each peak area of analyte/area IS (ceramide C12:0, 200 pmol) with calibration curve of each sphingolipids.

### Langendorff *ex vivo* functional assays

Hearts were excised from anesthetized rats; the aorta was immediately cannulated on the Langendorff perfusion system and perfused with 15 ml/min fix-flow modified HEPES-Krebs solution (pH 7.3 at 37°C). A latex balloon was inserted into the left ventricle, inflated (minimum of 10 mmHg), and connected to a pressure transducer (Bridge Amp/Power Lab 8/35; AD instruments) to monitor cardiac performance. After heart rate stabilization, hearts were perfused for 2 hours at constant flow with 30 ml of oxygenated modified HEPES-Krebs solution supplemented with 5 x 10^10^ EV 29. Heart rate and end-diastolic pressure were constantly monitored. Recirculating perfusate samples were collected at regular time points to measure cardiac Troponin I release (High Sensitivity Rat Cardiac Troponin-I ELISA, Life Diagnostics) and LDH release (cyQUANT LDH cytotoxicity assay kit, Invitrogen).

### Neonatal rat ventricular cardiomyocyte isolation and culture

1-3 days old neonatal rats were used as organ donors for *in vitro* neonatal cardiomyocyte cell cultures (NRVM). Pups were decapitated using sterile scissors, and the chest was opened along the sternum to allow access to the chest cavity and the heart. Hearts were extracted from the body and transferred immediately into plates containing 1X ADS Buffer, on ice. After atria removal, hearts were processed for CM isolation as previously described 59.

### Cytosolic and nuclear fractionation, total protein isolation and western blot analysis

Cells were centrifuged at 6900 rpm and pellets were suspended in 100μl cytosolic lysis buffer (20mM Tris-HCl pH7,8, 50mM KCl, 0.1mM DTT, 1mM PMSF), supplemented with protease inhibitors (SIGMA FAST TM Protease Inhibitor Tablets, Sigma), and vortexed at maximum speed for 30sec. Cytosolic protein fractions were collected after centrifugation at 14100rpm for 30 sec at 4°C. Nuclear pellets were suspended in 50μl nuclear lysis buffer (20mM Tris-HCl pH7,8, 50mM KCl, 0.1mM DTT, 1mM PMSF, 1% NP40) supplemented with protease inhibitors (SIGMAFAST^TM^ Protease Inhibitor Tablets, Sigma), and vortexed at maximum speed for 15min. Nuclear samples were then pulse sonicated for 10sec and supernatants were collected after centrifugation at 14100 rpm for 20min at 4°C.

Total proteins were extracted by lysing exosomes or cells with ice-cold RIPA buffer (25mM Tris pH:7,4 , 150mM NaCl, 1mM EDTA, 1% Igepal CA630, 1% Na-deoxycholate, 1.01% sodim dodecyl sulfate; SDS) supplemented with protease inhibitors (SIGMAFAST^TM^ Protease Inhibitor Tablets, Sigma) for 30 min at 4°C under agitation. EV and cell lysates were centrifuged at 9600 rpm for 15min at 4°C and protein concentrations were determined using BCA kit (Sigma). Equal amount of total proteins were boiled with Laemmli SDS sample buffer 6X (0.375M Tris-HCl pH 6.8, 12% SDS, 60% glycerol, 0.6 M DTT, 20%(v/v) β-mercaptoethanol, 0.2% (w/v) bromophenol blue; VWR International), separated on 4-20% Mini-PROTEAN®TGX™ Precast Gel (Bio-Rad), and transferred onto PVDF membranes with a semi-dry transfer system (Bio-Rad). The membranes were first blocked for 1 hr in Odyssey Blocking Buffer (LI-COR Biosciences) diluted 1:1 in distilled water and supplemented with 0.2% Tween 20 (OBB-T), then incubated with the appropriate primary Ab diluted in OBB-T at 4°C overnight under gentle agitation. The membranes were then incubated with an IRDye® 680RD or 800CW goat anti-mouse or goat anti-rabbit secondary Ab (LI-COR Biosciences; 1:15000 dilution in OBB-T) at RT for 2 h. The infrared signal was detected using the Odyssey CLx Detection System (LI-COR Biosciences). M0 polarization was assessed with the following primary antibodies: iNOS (abcam #3523, 1:500), TLR4 (Santa Cruz #sc293072, 1:500), CD68 (abcam #31630, 1:100) and normalized for GAPDH (abcam #181602, 1:1000) protein level. NRVM nuclear and cytosolic fractions were probed with p-NF-kBp65 ab (abcam #86299, 1:1000) and normalized respectively for H3 Histone (abcam #176842, 1:1000) and GAPDH (Abcam #ab181602).

### *In-vitro* neonatal rat ventricular myocyte viability assay

To assess cytotoxic effects of EV, NRVM cells were cultured for 5 hours in serum-free medium. After starvation, NRVM were pre-treated with a TLR4 inhibitor (0.1μM TAK242, InvivoGen) for 90 min and EV were then added at 10^7^/cm^2^ concentration as determined by Nanosight. Number of particles to be used *in vitro* experiments were determined previously in a dose response experiments 60. 12h later, cells were stained with CellstainTM Double Stain kit (dojindo) for 30 min at 37°C. 4X images were acquired with fluorescence microscopy (Nikon Eclipse-Ti) and the number of PI positive cells were quantified using ImageJ software (NIH).

### Statistical analysis

IBM SPSS statistics 24 (IBM Corp, Armonk, NY) and GraphPad Prism V7.0 (Graphpad Software, San Diego, CA) were used for statistical analyses. The distribution of each variable was assessed by the Kolmogorov-Smirnov test. Variables were expressed as mean ± SEM (standard error of measurement) and analyzed by Student's T test or One-way ANOVA test with post-hoc Bonferroni tests for multiple comparisons. Two-way ANOVA tests with post-hoc Bonferroni tests for multiple comparisons were used to analyze echocardiographic recordings and data from *ex vivo* Langendorff experiments. Survival probability of *in vivo* experiments was assessed with Kaplan-Meier curves. P-values of less than 0.05 were considered significant.

Additional methods are provided in the online supplementary methods.

## Supplementary Material

Supplementary figures and tables.

## Abbreviations

AAT: Alpha-1 antitrypsin
CM: cardiomyocytes
DAMPs: damage-associated molecular patterns
EDP: end diastolic pressure
EV: extracellular vesicles
EV pre-MI: plasma-derived EV before myocardial infarction
EV post-MI: plasma-derived EV after myocardial infarction
FBS: fetal bovine serum
ILV: intraluminal vesicles
I/R: ischemia-reperfusion
LAD: permanent coronary artery ligation
LVDV: LV end-diastolic volume
LVEF: Left ventricular ejection fraction
LVSV: LV end-systolic volume
MΦ: macrophages
MI: myocardial infarction
MVB: multivesicular bodies
NLRs: nucleotide-binding oligomerization domain-like receptors
NRVM: neonatal rat ventricular myocytes
nSMases: neutral sphingomyelinase
NTA: nanoparticle tracking analysis
PRRs: pattern recognition receptors
STEMI: ST-segment elevation MI
Tau: constant of isovolumic LV pressure
TEM: transmission electron microscopy
TRLs: activation of toll-like receptor
