# Inflammatory extracellular vesicles prompt heart dysfunction via TLR4-dependent NF-κB activation: Erratum

**DOI:** 10.7150/thno.83071

**Published:** 2023-02-08

**Authors:** Vanessa Biemmi, Giuseppina Milano, Alessandra Ciullo, Elisabetta Cervio, Jacopo Burrello, Michele Dei Cas, Rita Paroni, Tiziano Tallone, Tiziano Moccetti, Giovanni Pedrazzini, Sarah Longnus, Giuseppe Vassalli, Lucio Barile

**Affiliations:** 1 Laboratory for Cardiovascular Theranostics, Cardiocentro Ticino Institute, Lugano, Switzerland; 2 Laboratory of Cellular and Molecular Cardiology, Cardiocentro Ticino Institute, Lugano, Switzerland; 3 Dept. Cœur-Vaisseaux, Centre Hospitalier Universitaire Vaudois, Lausanne, Switzerland; 4 Department of Health Sciences of the University of Milan, Milan, Italy; 5 Cell and Biomedical Technologies Unit Cardiocentro Ticino Institute, Lugano, Switzerland; 6 Department of Cardiology, Cardiocentro Ticino Institute, Lugano, Switzerland; 7 Department of Cardiovascular Surgery, Inselspital, Bern University Hospital, Bern, Switzerland; 8 Faculty of Biomedical Sciences, Università della Svizzera italiana, Lugano, Switzerland

The authors regret that the original version of above paper, unfortunately, contained three typos consisting of the incorrect spelling of “Toll-like receptor” acronym, TRL4 instead of TLR4. The correct spelling is shown below:

Title: “Inflammatory extracellular vesicles prompt heart dysfunction via TLR4-dependent NF-κB activation”

Introduction: “The systemic release of different subsets of cytokines and proteins by polarized M1/M2 macrophages implies the activation of toll-like receptor (TLR) and subsequent NF-κB signalling cascades [8].”

Methods: “transmission electron microscopy; TLR: activation of toll-like receptor”

The correction made in this erratum does not concern any published data. The authors apologize for any inconvenience that the errors may have caused.

