# Supplementary material for: Inflammatory extracellular vesicles prompt heart dysfunction via TRL4-dependent NF-κB activation
Source: Theranostics. 2020 Feb 3;10(6):2773–90. doi: 10.7150/thno.39072 (PMC7052909; doi:10.7150/thno.39072)
Supplement: Supplementary file 1 — Supplementary figures and tables. [file thnov10p2773s1.pdf]

**Supplemental methods**

*Transmission electron microscopy-negative staining*

EV derived from 100ul of rat plasma were diluted 1:100 and absorbed on a glow- discharged carbon coated formvar nickel grid and negatively stained with uranyl acetate. Representative plasma derived EV were acquired and examined by Talos L120C electron microscope at 120kV.

*RNA extraction, reverse transcription and real-time PCR*

48hrs after polarization, BMDM were collected and total RNA extracted with TRI-Reagent (Sigma) and chloroform following manufacturer's instructions. 500ng of total RNA was reverse transcribed using GoScript™ Reverse Transcription System (Promega Madison) following manufacturer's instructions. mRNA EV content and BMDM polarization was assessed by real-time PCR using SYBR Green labeling protocol (BioRad) following the manufacturer's instructions.

mRNA content of EV pre-MI, EV post MI Vehicle and EV Post MI GW4869 were determined by real time PCR for iNOS (Fw primer: AGTCCTCTTTGCTACTGAGACAAGG, Re primer: CACCACCAGCAGTAGTTGTTC), INFγ ( Fw primer: CAAGTTCGAGGTGAACAACCC, Re primer GGCACACTCTCTACCCCAGA) , IL1α (Fw primer: GGTGGTGTCAGCAACATCAAA, Re primer TCTGGGTTGGATGGTCTCTTCT), IL1β (Fw primer: TCCTCTGTGACTCGTGGGAT, Re primer: TGGAGAATACCACTTGTTGGCT ), Rantes (Fw primer: ATATGGCTCGGACACCACTC, Re primer: GTGACAAAGACGACTGCAAGGT) and IL6 (Fw primer: GCAAGAGACTTCCAGCCAGT, Re primer: TGCACAACTCTTTTCTCATTTCCA).

Amplification and detection of specific products were performed in triplicate using CFX Connect™ Real- Time PCR Detection System (Bio-Rad). The threshold cycle (Ct) of each gene was defined and normalized to the control GAPDH (Fw primer: TGCACCACCAACTGCTTAGC, Re primer: GGCATGGACTGTGGTCATGAG).

BMDM pro inflammatory M1 phenotype was characterized by upregulation of iNOS (Fw primer: AGTCCTCTTTGCTACTGAGACAAGG, Re primer: CACCACCAGCAGTAGTTGTTC), TLR4 (Fw primer: TCTGCCCTGCCACCATTTAC, Re primer: GAAGTACCTCTATGCAGGGATTCA) and TNFα (Fw primer: ATTGTGGCTCTGGGTCCAAC, Re primer: CGCAATCCAGGCCACTACTT) whereas M2 profile by CD206 (Fw primer: GAGGACTGCGTGGTGATGAA, Re primer: CATGCCGTTTCCAGCCTTTC) and Arginase1 (Fw primer: ACAAGACAGGGCTACTTTCAGG, Re primer: ACAAGACAAGGTCAACGCCA) over-expression. Amplification and detection of specific products were performed in triplicate using CFX Connect™ Real- Time PCR Detection System (Bio-Rad). The threshold cycle (Ct) of each gene was defined and normalized to the control GAPDH (Fw primer: TGCACCACCAACTGCTTAGC, Re primer: GGCATGGACTGTGGTCATGAG) and data were shown as $2^{-\Delta\Delta Ct}$.

*Immunofluorescence assays*

Cells and hearts sections were fixed with 4% paraformaldehyde solution, permeabilized with 0.5% TritonX for 30 minutes and blocked with 2% bovine serum albumin (BSA) for 30 minutes at 37°C. Cells were then incubated with primary antibodies against NF-κBp65 (Thermofisher #510500, 1:100) and, as marker for NRVM, α sarcomeric actinin (Abcam #ab9465, 1:100) diluted in PBS with 0.1%Tween20 and 0.2% BSA overnight at 4°C. Alexa Fluor secondary antibodies (Life Technologies, 1:1000) were used for detection and DAPI staining was used for nuclear localization. Images were acquired by the C2 Plus confocal microscopy system (Nikon) and analysed using ImageJ software. To evaluate heart macrophage infiltration, six sections for each heart were stained with anti-CD68 (abcam #ab31630, 1:100). Quantitative analyses of fluorescent signal intensity and the number of positive cells were quantified using ImageJ software (NIH).

Tissue and heart sections apoptosis were stained with anti-cleaved caspase 3 (Cell Signaling #9664, 1:100), anti-cleaved caspase 7 (Cell Signaling #8438, 1:100), α sarcomeric actinin (Abcam #ab9465,

1:100 and DAPI. Fluorescence signal intensity was quantified using Image J software and normalized to the number of positive cells.

*Masson-trichrome Histology*

After fixation with 4% paraformaldehyde, hearts were cryopreserved in optimal cutting temperature (OCT) medium at -80°C and cut in 8 μm sections. Six sections distributed from the cardiac base to apex were stained for Masson-trichrome (HT15 Trichrome Stain Kit, Sigma) for measurement of scar size. For each heart section, scar area was determined by tracing infarct border zone using ImageJ and values were averaged.

*Hypoxia In-vitro neonatal rat ventricular myocyte viability assay*

To assess cytotoxic effects of macrophages derived EV under hypoxia condition, NRVM cells were cultured for 5 hours in serum-free medium. After starvation, NRVM were treated with $10^7/cm^2$ BMDM-EV as determined by Nanosight and incubated for 12 h in hypoxia condition (1% $O_2$). Cells were than stained with CellstainTM Double Stain kit (dojindo) for 30 min at 37°C under normoxia condition. 4X and 10X images were acquired with fluorescence microscopy (Nikon Eclipse-Ti) and the number of PI positive cells were quantified using ImageJ software (NIH).

*Extracellular vesicles glycine acid treatment*

To remove extracellular vesicles surface associated protein, equal amount of isolated EV for each condition was incubated with 100mM glycine pH2.5 for 5 minutes at RT. After incubation, pH was neutralized with 2M Tris pH 8.00. To avoid any interference of glycine solution, EV were washed in 3 ml of PBS 1X and pelleted at 100000g (3h).

**Supplemental Figure**

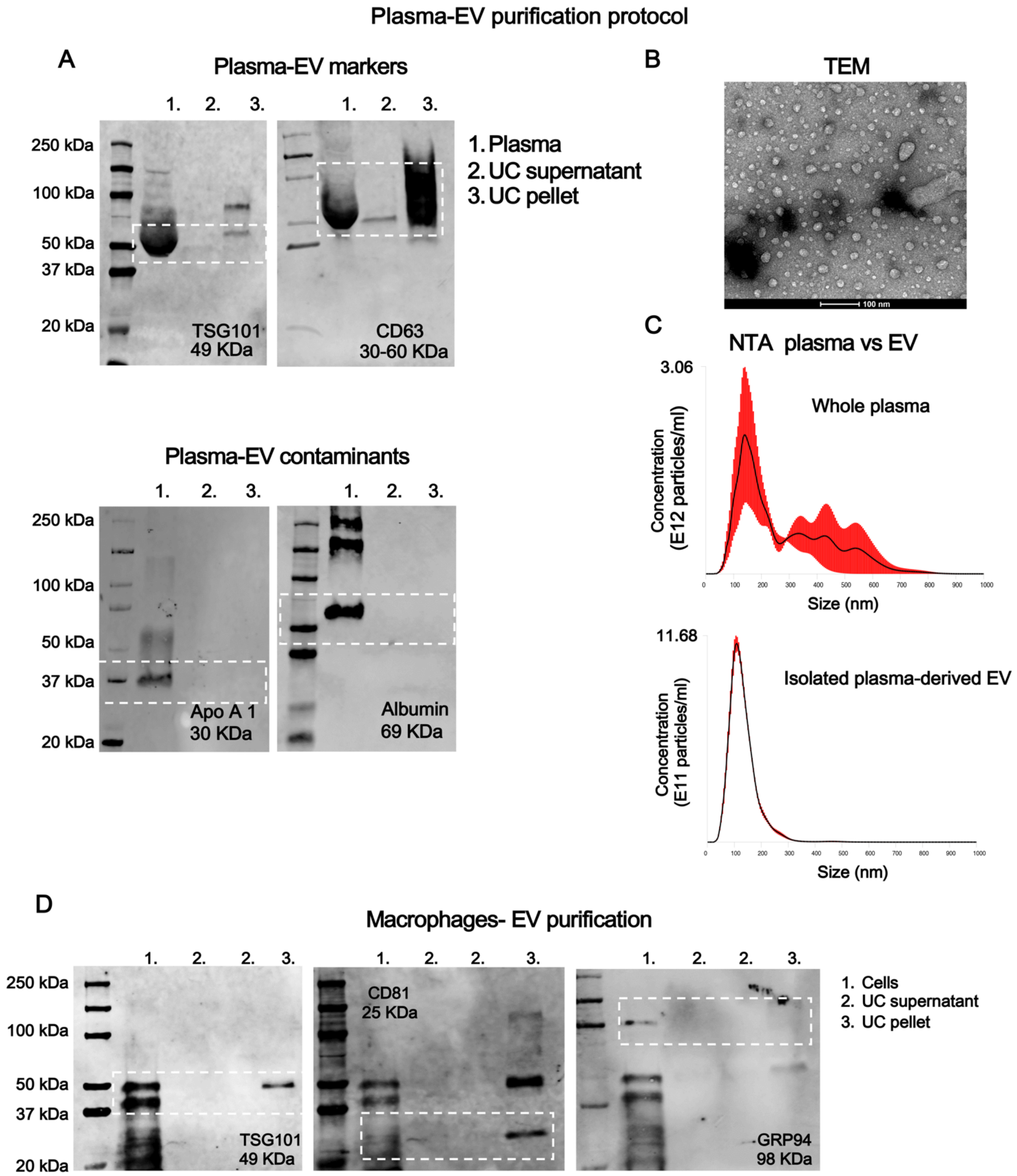


**Figure S1. BMDM and Plasma-EV characterization. (A)** Western blotting analysis of whole plasma and plasma derived EV of specific exosomal markers TSG101 and CD63 and plasma contaminants ApoA1 and Albumin not present in EV preparations. **(B)** Representative plasma derived EV transmission electron microscopy**. (C)** Dynamic light scatter of particle size and

concentration of whole plasma and plasma derived EV. Red lines represent standard deviations. **(D)** BMDM derived EV characterization was assessed by the presence of TSG101 and CD81 and the absence of contaminants GRP94.

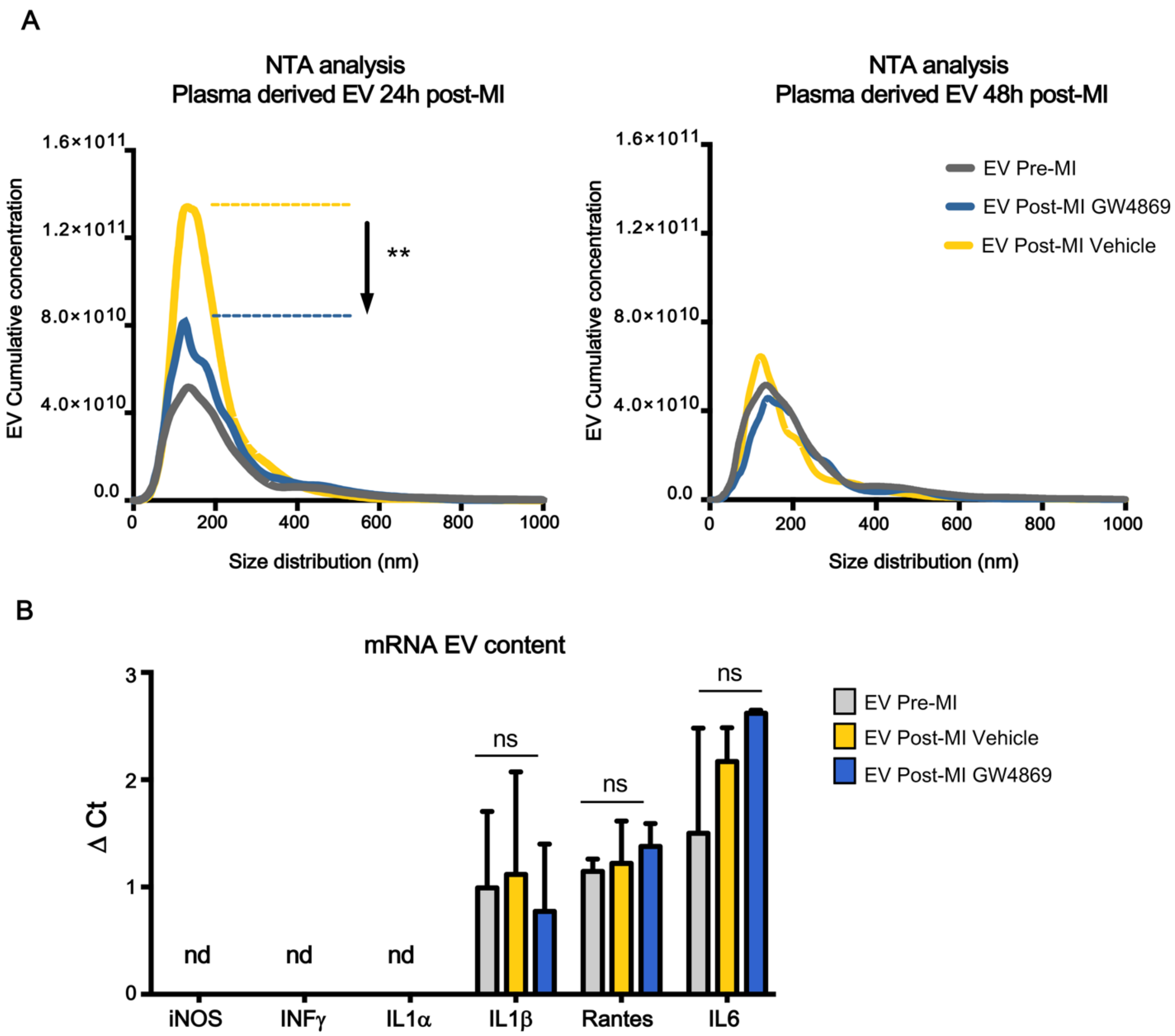


**Figure S2. EV mRNA content and concentration after MI. (A)** NTA analysis of plasma extracellular vesicles concentration 24 hours and 48 hours after myocardial infarction induction. Cumulative curves showing concentration (y-axis) and size distribution (x-axis) **(B)** EV mRNA content analysis. Data are reported as ΔCt and normalized by GAPDH. All data are presented as mean ± SEM and analysed by one way ANOVAs with post-hoc multiple comparisons using Bonferroni correction. Mean, SEM and statistics are reported in full in Table S9.

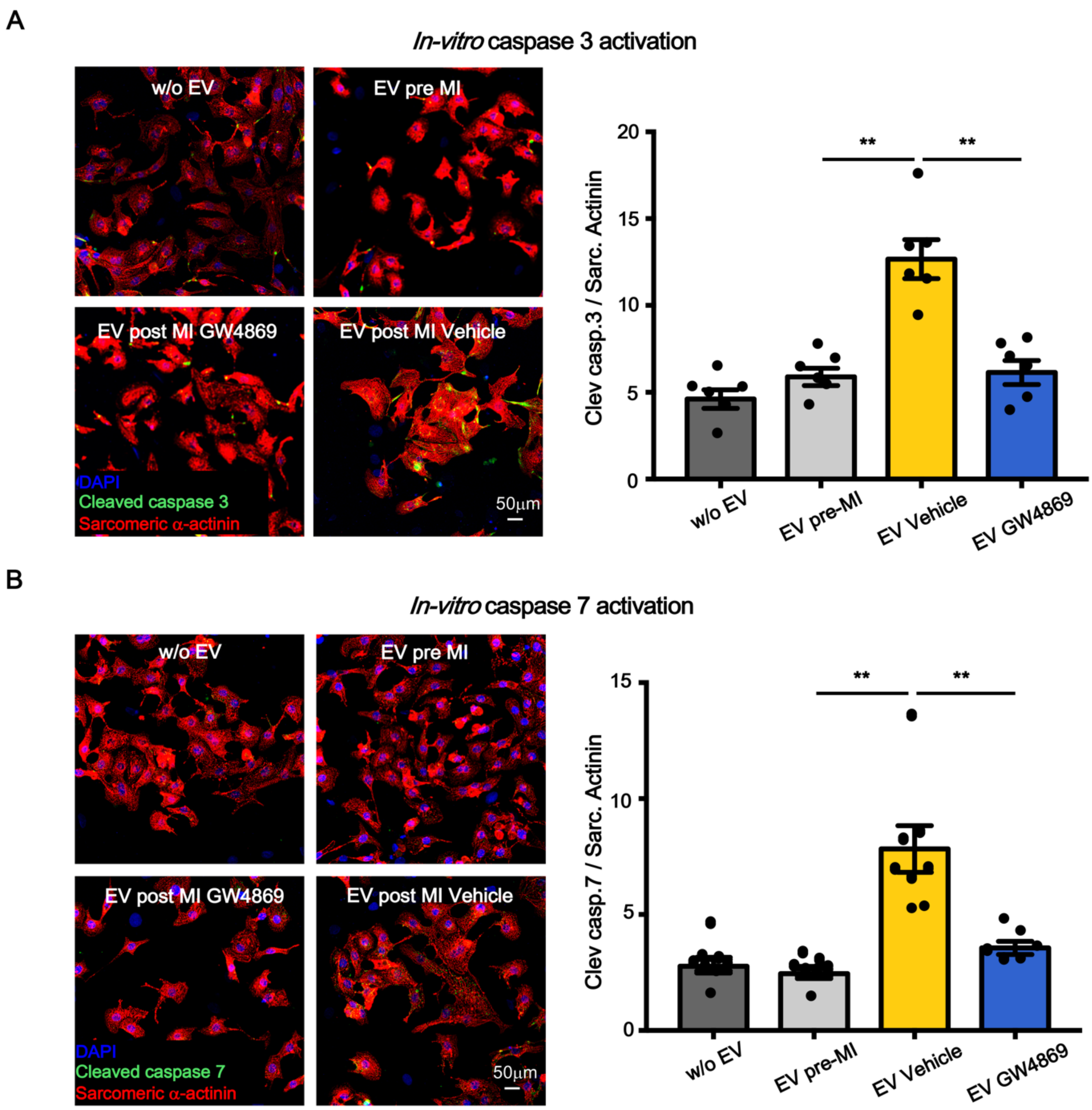


**Figure S3. *In-vitro* cleaved caspase 3 and 7 activation. (A)** Representative images and quantification of caspase 3 activation (cleaved caspase 3) in NRVM after the treatment with EV pre-MI, EV Vehicle and EV GW4869. **(B)** Representative images and quantification of caspase 7 activation (cleaved caspase 7) in NRVM treated with EV pre-MI, EV Vehicle and EV GW4869. Quantification analysis of fluorescence signal intensity are normalized to the number of cardiomyocytes. All data are presented as mean ± SEM and analysed by one way ANOVA with post-hoc multiple comparisons using Bonferroni correction. Mean, SEM and statistics are reported in full in Table S10.

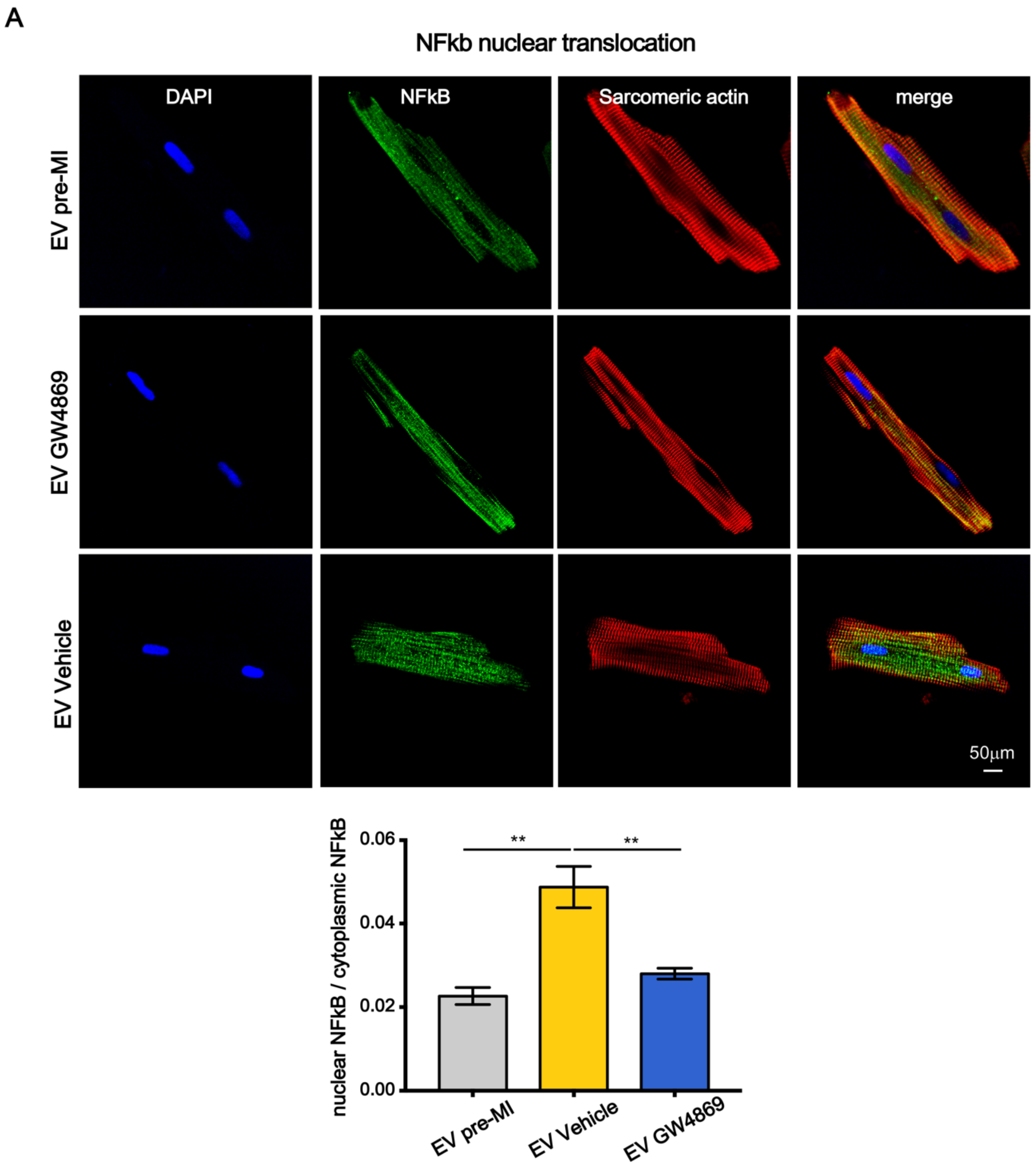


**Figure S4. NFκB nuclear translocation in isolated adult cardiomyocytes. (A)** Representative images and quantification of NFκB nuclear translocation in adult cardiomyocytes isolated from healthy hearts after 90min of EV Langendorff perfusion. EV pre-MI or EV vehicle or EV GW4869 were added to the perfusate solution. DAPI mask was used to detect NFκB nuclear fraction. All data are presented as mean ± SEM and analysed by one way ANOVAs with post-hoc multiple comparisons using Bonferroni correction. Mean, SEM and statistics are reported in full in Table S11.

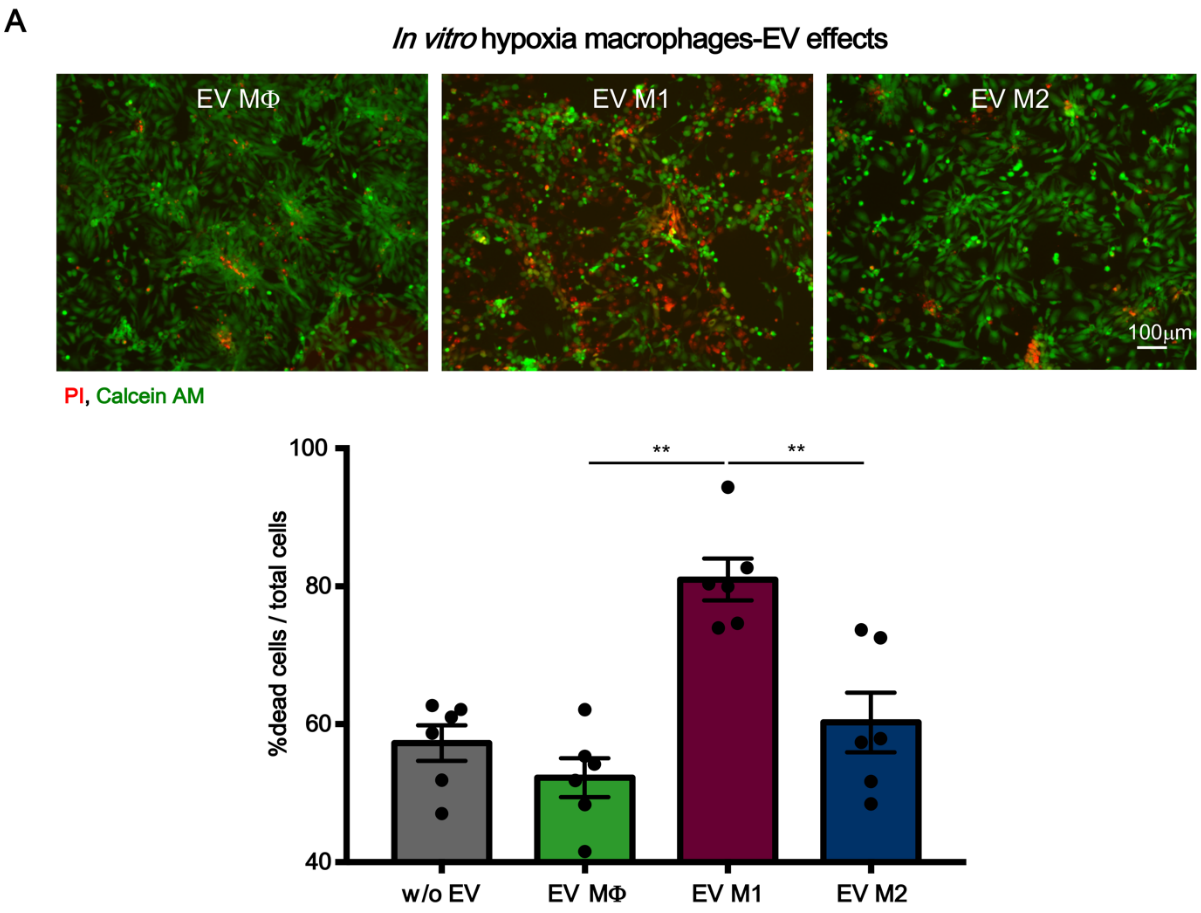


**Figure S5. *In-vitro* macrophages derived EV cytotoxic effect under hypoxia. (A)** Quantification of BMDM derived EV cytotoxicity on NRVM simultaneously exposed to hypoxia and EV treatment. All data are presented as mean ± SEM and analysed by one way ANOVAs with post-hoc multiple comparisons using Bonferroni correction. Mean, SEM and statistics are reported in full in Table S12.

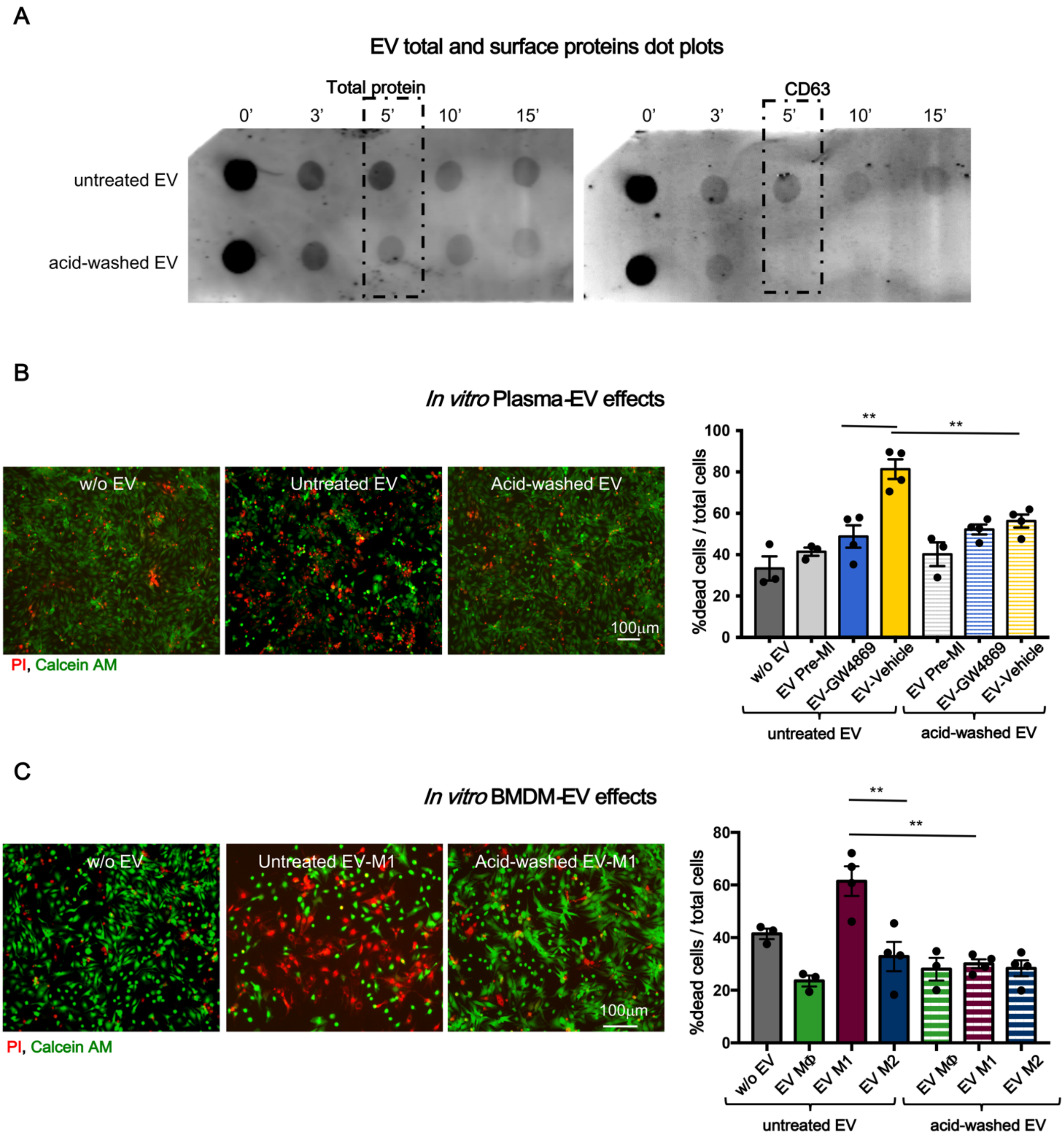


**Figure S6. Vesicles cytotoxic effects are mediated by EV surface proteins. (A)** Dot blot analysis of untreated EV and acid-washed EV of total protein and specific EV surface protein CD63. **(B)** Representative images and quantification of plasma derived EV cytotoxicity on NRVM exposed to untreated-EV or surface associated protein depleted-EV (acid-washed EV). **(C)** Representative images and quantification of BMDM derived EV cytotoxicity on NRVM exposed to untreated-EV or surface associated protein depleted-EV (acid-washed EV). All data are presented as mean ± SEM and analysed by one way ANOVAs with post-hoc multiple comparisons using Bonferroni correction. Mean, SEM and statistics are reported in full in Table S13.

**Statistical Analyses -** Each variable is expressed by mean ± SEM. Data are analyzed by ANOVA one-way tests and Bonferroni post-hoc test, except if differently indicated. Differences were considered significant for $p < 0.05$.

## Table S1 – Plasma-EV analysis

| Variable | w/o EV | EV Pre-MI | EV Post-MI | Overall *P*-value | Pairwise Comparisons | | |
|---|---|---|---|---|---|---|---|
| | | | | | w/o vs. Pre-MI | w/o vs. Post-MI | Pre-MI vs. Post-MI |
| Dead cells / total cells (%) | 24.3 ± 3.15 | 31.9 ± 4.12 | 66.7 ± 2.83 | **< 0.001** | 0.536 | **< 0.001** | **< 0.001** |

w/o: without; EV: Extracellular Vesicles; MI: Myocardial Infarction.

## Table S2 – Inhibition of EV release regulates inflammation in heart and reduces inflammatory EV after MI

| Variable | Vehicle | GW4869 | *P*-value |
|---|---|---|---|
| Cer C24:0 | 2.44 ± 0.114 | 2.24 ± 0.108 | 0.220 |
| Cer C24:1 | 3.13 ± 0.156 | 2.75 ± 0.159 | 0.108 |
| Cer C22:0 | 2.43 ± 0.161 | 2.03 ± 0.172 | 0.110 |
| Cer C20:0 | 1.31 ± 0.115 | 1.19 ± 0.126 | 0.495 |
| Cer C18:0 | 1.63 ± 0.156 | 2.15 ± 0.205 | 0.064 |
| Cer C18:1 | 1.00 ± 0.110 | 0.67 ± 0.113 | 0.053 |
| Cer C16:0 | 2.14 ± 0.051 | 2.01 ± 0.048 | 0.081 |
| Cer C14:0 | 1.17 ± 0.117 | 1.00 ± 0.110 | 0.304 |
| Cer TOT (pmol) | 2.23 ± 0.136 | 2.09 ± 0.134 | 0.474 |

| Variable | EV Pre-MI | EV Post-MI Vehicle | EV Post-MI GW4869 | Overall *P*-value | Pairwise Comparisons | | |
|---|---|---|---|---|---|---|---|
| | | | | | **Pre-MI vs. Vehicle** | **Pre-MI vs. GW4869** | **Vehicle vs. GW4869** |
| NTA – Cumulative concentration (AUC) | 0.19 ± 0.038 | 0.61 ± 0.095 | 0.26 ± 0.039 | **< 0.001** | **< 0.001** | 1.000 | **0.002** |

| Variable | EV Pre-MI | EV Post-MI Vehicle | EV Post-MI GW4869 | Overall *P*-value | Pairwise Comparisons | | |
|---|---|---|---|---|---|---|---|
| | | | | | Pre-MI vs. Vehicle | Pre-MI vs. GW4869 | Vehicle vs. GW4869 |
| iNOS / Tsg101 (a.u.) | 0.9 ± 0.15 | 3.7 ± 0.52 | 1.5 ± 0.14 | **< 0.001** | **< 0.001** | 0.738 | **0.001** |
| CD68 / Tsg101 (a.u.) | 22 ± 3,8 | 110 ± 8,8 | 68 ± 4,2 | **< 0.001** | **< 0.001** | **0.001** | **0.003** |

| Variable | EV Pre-MI | EV Post-MI Vehicle | EV Post-MI GW4869 | Overall *P*-value | Pairwise Comparisons | | |
|---|---|---|---|---|---|---|---|
| | | | | | Pre-MI vs. Vehicle | Pre-MI vs. GW4869 | Vehicle vs. GW4869 |
| IL-4 (FC) | 0.86 ± 0.10 | 0.98 ± 0.04 | 1.42 ± 0.09 | **< 0.001** | 0.756 | **< 0.001** | **0.002** |
| IL-6 (FC) | 0.92 ± 0.05 | 1.03 ± 0.05 | 1.21 ± 0.02 | **< 0.001** | 0.279 | **< 0.001** | **0.018** |
| IL-10 (FC) | 0.87 ± 0.05 | 0.87 ± 0.04 | 1.06 ± 0.01 | **0.002** | 1.000 | **0.006** | **0.006** |
| IL-1α (FC) | 1.12 ± 0.06 | 1.50 ± 0.03 | 1.26 ± 0.05 | **< 0.001** | **< 0.001** | 0.143 | **0.003** |
| IL-1β (FC) | 1.00 ± 0.05 | 1.41 ± 0.11 | 1.12 ± 0.06 | **0.004** | **0.004** | 0.959 | **0.045** |
| Rantes (FC) | 1.22 ± 0.05 | 2.02 ± 0.13 | 1.52 ± 0.11 | **< 0.001** | **< 0.001** | 0.145 | **0.006** |

| Variable | Sham | Vehicle | GW4869 | Overall *P*-value | Pairwise Comparisons | | |
|---|---|---|---|---|---|---|---|
| | | | | | Sham vs. Vehicle | Sham vs. GW4869 | Vehicle vs. GW4869 |
| Tissue CD68$^+$ cells (n°/mm$^2$) | 1.1 ± 0.42 | 70.7 ± 5.18 | 46.0 ± 7.92 | **< 0.001** | **< 0.001** | **< 0.001** | **0.020** |
| Tissue TNFα (pg/500 µg protein) | 25.0 ± 0.89 | 89.1 ± 13.09 | 44.6 ± 5.77 | **< 0.001** | **< 0.001** | 0.271 | **0.003** |

Cer: ceramide; EV: Extracellular Vesicles; MI: Myocardial Infarction a.u.: arbitrary unit; FC: fold change; AUC: area under the curve.

## Table S3 – *In vivo* inhibition of EV release mitigates myocardial dysfunction after permanent coronary artery ligation

| Variable | | Vehicle | GW4869 | *P*-value |
|---|---|---|---|---|
| LV Ejection Fraction (%) | Before-MI | 81.0 ± 2.02 | 80.8 ± 2.16 | 1.000 |
| | Day 1 | 53.8 ± 3.62 | 54.0 ± 3.07 | 1.000 |
| | Day 7 | 61.6 ± 4.13 | 68.1 ± 3.40 | 0.642 |
| | Day 28 | 52.2 ± 5.49 | 70.0 ± 4.15 | **0.004** |
| LV end-systolic Volume (uL) | Before-MI | 45.0 ± 5.01 | 57.9 ± 7.98 | 1.000 |
| | Day 1 | 182.0 ± 18.19 | 159.6 ± 20.13 | 1.000 |
| | Day 7 | 179.4 ± 26.19 | 128.7 ± 17.75 | 0.341 |
| | Day 28 | 273.9 ± 32.97 | 128.1 ± 21.86 | **< 0.001** |
| LV end-diastolic Volume (uL) | Before-MI | 288.4 ± 15.10 | 304.7 ± 7.80 | 1.000 |
| | Day 1 | 392.5 ± 20.47 | 366.4 ± 20.71 | 1.000 |
| | Day 7 | 433.3 ± 32.13 | 401.3 ± 28.55 | 1.000 |
| | Day 28 | 564.0 ± 26.74 | 421.4 ± 28.99 | **0.001** |

| Variable | Sham | Vehicle | GW4869 | Overall *P*-value | Pairwise Comparisons | | |
|---|---|---|---|---|---|---|---|
| | | | | | Sham vs. Vehicle | Sham vs. GW4869 | Vehicle vs. GW4869 |
| Tau index (ms) | 10.3 ± 0.14 | 13.7 ± 0.70 | 10.6 ± 0.80 | **0.009** | **0.022** | 1.000 | **0.019** |
| LV systolic pressure (mmHg) | 119.6 ± 4.14 | 102.6 ± 3.65 | 119.9 ± 3.62 | **0.011** | **0.031** | 1.000 | **0.020** |
| dP/dt max (mmHg/s) | 8841.8 ± 1097.72 | 6549.0 ± 306.33 | 9166.9 ± 543.05 | **0.030** | 0.105 | 1.000 | **0.042** |
| dP/dt min (mmHg/s) | -7778.3 ± 755.44 | -5243.9 ± 602.9 | -8574.6 ± 692.6 | **0.011** | 0.078 | 1.000 | **0.013** |
| Scar size / Total section area (%) | N.A. | 24.0 ± 4.49 | 7.4 ± 2.10 | **0.006** | N.A. | N.A. | N.A. |

Echocardiographic parameters were analyzed by two-way ANOVA test. LV: Left Ventricle.

## Table S4 – *In vivo* inhibition of EV release mitigates myocardial dysfunction after ischemia-reperfusion injury

| Variable | | Vehicle | GW4869 | *P*-value |
|---|---|---|---|---|
| LV Ejection Fraction (%) | Before-MI | 84.5 ± 1.13 | 80.8 ± 2.00 | 1.000 |
| | Day 1 | 61.4 ± 2.70 | 62.9 ± 4.60 | 1.000 |
| | Day 7 | 63.5 ± 2.75 | 75.0 ± 2.17 | **0.019** |
| | Day 28 | 61.7 ± 2.84 | 75.8 ± 2.17 | **0.002** |
| LV end-systolic Volume (uL) | Before-MI | 45.0 ± 5.01 | 62.4 ± 8.77 | 1.000 |
| | Day 1 | 112.8 ± 15.33 | 107.5 ±14.04 | 1.000 |
| | Day 7 | 147.0 ± 18.98 | 94.6 ± 8.69 | **0.047** |
| | Day 28 | 196.8 ± 23.41 | 112.7 ± 14.88 | **< 0.001** |
| LV end-diastolic Volume (uL) | Before-MI | 288.4 ± 15.11 | 319.6 ± 17.62 | 1.000 |
| | Day 1 | 299.2 ± 23.57 | 284.8 ± 17.61 | 1.000 |
| | Day 7 | 405.2 ± 28.44 | 399.5 ± 26.45 | 1.000 |
| | Day 28 | 494.4 ± 29.13 | 469.1 ± 28.92 | 1.000 |

| Variable | Sham | Vehicle | GW4869 | Overall *P*-value | Pairwise Comparisons | | |
|---|---|---|---|---|---|---|---|
| | | | | | Sham vs. Vehicle | Sham vs. GW4869 | Vehicle vs. GW4869 |
| Tau index (ms) | 10.3 ± 0.13 | 13.32 ± 0.45 | 12.6 ± 0.23 | **< 0.001** | **< 0.001** | **0.001** | 0.360 |
| LV systolic pressure (mmHg) | 119.6 ± 4.14 | 113.2 ± 1.50 | 125.9 ± 3.99 | **0.037** | 0.760 | 0.744 | **0.034** |
| dP/dt max (mmHg/s) | 8841.8 ± 1097.73 | 7989.8 ± 474.46 | 7785.1 ± 339.90 | 0.517 | N.A. | N.A. | N.A. |
| dP/dt min (mmHg/s) | -7778.3 ± 755.44 | -7839.1 ± 489.61 | -7205.9 ± 388.22 | 0.651 | N.A. | N.A. | N.A. |
| Scar size / Total section area (%) | N.A. | 15.3 ± 1.72 | 7.1 ± 0.90 | **0.001** | N.A. | N.A. | N.A. |

Echocardiographic parameters were analyzed by ANOVA two-way test. LV: Left Ventricle.

## Table S5 – *Ex vivo* plasma derived EV cytotoxicity in cardiomyocytes

| Langendorff EDP (mmHg) | w/o EV | EV Pre-MI | EV Post-MI GW4869 | EV Post-MI Vehicle | Overall *P*-value | Pairwise Comparisons | | | | | |
|---|---|---|---|---|---|---|---|---|---|---|---|
| | | | | | | w/o EV vs. EV Pre-Mi | w/o EV vs. EV GW4869 | w/o EV vs. EV Vehicle | EV Pre-MI vs. EV GW4869 | EV Pre-MI vs. EV Vehicle | EV GW4869 vs. Vehicle |
| 0 min | 9.9 ± 1.36 | 6.5 ± 0.46 | 7.6 ± 0.89 | 9.5 ± 0.88 | **< 0.001** | 1.000 | 1.000 | 1.000 | 1.000 | 1.000 | 1.000 |
| 10 min | 8.0 ± 0.97 | 5.5 ± 0.69 | 6.0 ± 0.67 | 8.3 ± 0.60 | | 1.000 | 1.000 | 1.000 | 1.000 | 1.000 | 1.000 |
| 20 min | 9.0 ± 0.81 | 9.8 ± 0.75 | 7.4 ± 0.32 | 13.7 ± 1.46 | | 1.000 | 1.000 | 0.875 | 1.000 | 1.000 | 0.248 |
| 30 min | 10.6 ± 0.49 | 12.1 ± 1.19 | 8.4 ± 0.41 | 18.7 ± 2.09 | | 1.000 | 1.000 | 0.073 | 1.000 | 0.316 | **0.006** |
| 40 min | 10.8 ± 0.90 | 13.7 ± 1.33 | 10.9 ± 0.62 | 21.2 ± 2.89 | | 1.000 | 1.000 | **0.008** | 1.000 | 0.168 | **0.005** |
| 50 min | 11.6 ± 0.63 | 14.5 ± 1.24 | 15.2 ± 1.54 | 31.5 ± 3.30 | | 1.000 | 1.000 | **< 0.001** | 1.000 | **< 0.001** | **< 0.001** |
| 60 min | 15.8 ± 2.32 | 20.3 ± 2.46 | 17.6 ± 2.46 | 36.0 ± 2.92 | | 1.000 | 1.000 | **< 0.001** | 1.000 | **< 0.001** | **< 0.001** |
| 70 min | 17.0 ± 2.47 | 27.9 ± 1.68 | 19.7 ± 3.16 | 42.8 ± 1.01 | | **0.002** | 1.000 | **< 0.001** | 0.108 | **< 0.001** | **< 0.001** |
| 80 min | 20.2 ± 2.70 | 28.6 ± 1.30 | 23.7 ± 3.67 | 50.5 ± 0.87 | | 0.130 | 1.000 | **< 0.001** | 0.917 | **< 0.001** | **< 0.001** |
| 90 min | 26.8 ± 3.36 | 34.4 ± 2.02 | 28.1 ± 4.02 | 54.4 ± 2.63 | | 0.210 | 1.000 | **< 0.001** | 0.381 | **< 0.001** | **< 0.001** |

| Perfusate Cardiac Troponin I (ng tot) | w/o EV | EV Pre-MI | EV Post-MI GW4869 | EV Post-MI Vehicle | Overall *P*-value | Pairwise Comparisons | | | | | |
|---|---|---|---|---|---|---|---|---|---|---|---|
| | | | | | | w/o EV vs. EV Pre-Mi | w/o EV vs. EV GW4869 | w/o EV vs. EV Vehicle | EV Pre-MI vs. EV GW4869 | EV Pre-MI vs. EV Vehicle | EV GW4869 vs. Vehicle |
| 0 min | 0.0 ± 0.00 | 7.9 ± 5.58 | 27.8 ± 13.63 | 34.4 ± 26.16 | **< 0.001** | 1.000 | 1.000 | 1.000 | 1.000 | 1.000 | 1.000 |
| 10 min | 0.0 ± 0.00 | 91.7 ± 31.88 | 178.3 ± 96.21 | 503.8 ± 95.84 | | 1.000 | 1.000 | 1.000 | 1.000 | 1.000 | 1.000 |
| 20 min | 40.1 ± 28.38 | 258.3 ± 74.63 | 485.4 ± 136.50 | 1349.0 ± 374.01 | | 1.000 | 1.000 | 0.070 | 1.000 | 0.205 | 0.371 |
| 40 min | 512.8 ± 81.28 | 775.4 ± 119.17 | 1147.6 ± 279.83 | 2942.8 ± 443.08 | | 1.000 | 1.000 | **< 0.001** | 1.000 | **< 0.001** | **0.001** |
| 50 min | 816.4 ± 168.19 | 1562.7 ± 365.72 | 1992.3 ± 344.59 | 3413.8 ± 529.87 | | 1.000 | 0.111 | **< 0.001** | 1.000 | **0.002** | **0.020** |
| 60 min | 1099.7 ± 177.06 | 2148.4 ± 528.51 | 2538.7 ± 413.76 | 4359.9 ± 414.06 | | 0.413 | **0.033** | **< 0.001** | 1.000 | **< 0.001** | **0.001** |
| 80 min | 2232.8 ± 244.16 | 3014.1 ± 375.55 | 3858.9 ± 493.63 | 5633.8 ± 349.69 | | 1.000 | **0.013** | **< 0.001** | 0.615 | **< 0.001** | **0.002** |

| Perfusate LDH (U tot) | w/o EV | EV Pre-MI | EV Post-MI GW4869 | EV Post-MI Vehicle | Overall *P*-value | Pairwise Comparisons | | | | | |
|---|---|---|---|---|---|---|---|---|---|---|---|
| | | | | | | w/o EV vs. EV Pre-Mi | w/o EV vs. EV GW4869 | w/o EV vs. EV Vehicle | EV Pre-MI vs. EV GW4869 | EV Pre-MI vs. EV Vehicle | EV GW4869 vs. Vehicle |
| 0 min | 547.5 ± 2.91 | 610.6 ± 46.82 | 566.3 ± 1.70 | 559.2 ± 10.37 | | 0.377 | 1.000 | 1.000 | 1.000 | 0.841 | 1.000 |
| 10 min | 575.0 ± 6.18 | 742.3 ± 99.47 | 606.1 ± 21.45 | 609.2 ± 6.67 | | 0.149 | 1.000 | 1.000 | 0.431 | 0.466 | 1.000 |
| 20 min | 633.9 ± 22.81 | 822.0 ± 95.01 | 709.1 ± 32.36 | 712.1 ± 44.14 | | 0.140 | 1.000 | 1.000 | 1.000 | 1.000 | 1.000 |
| 40 min | 740.8 ± 48.48 | 933.8 ± 90.07 | 836.6 ± 65.87 | 996.5 ± 90.67 | **< 0.001** | 0.471 | 1.000 | 0.151 | 1.000 | 1.000 | 0.944 |
| 50 min | 786.3 ± 70.14 | 921.3 ± 78.56 | 921.9 ± 144.75 | 1157.7 ± 106.42 | | 1.000 | 1.000 | 0.116 | 1.000 | 0.787 | 0.792 |
| 60 min | 843.4 ± 68.96 | 1001.2 ± 86.25 | 1052.1 ± 145.39 | 1366.2 ± 93.80 | | 1.000 | 0.912 | **< 0.001** | 1.000 | **0.001** | **0.024** |
| 80 min | 986.5 ± 50.63 | 1022.8 ± 60.13 | 1247.3 ± 142.05 | 1589.4 ± 43.47 | | 1.000 | 0.216 | **< 0.001** | 0.472 | < **0.001** | **0.011** |

Data were analyzed by ANOVA two-way tests and Bonferroni post-hoc tests. w/o: without; EV: Extracellular Vesicles; MI: Myocardial Infarction; EDP: end-diastolic pressure.

| Variable | w/o EV | EV Pre-MI | EV Post-MI GW4869 | EV Post-MI Vehicle | Overall *P*-value | Pairwise Comparisons | | | | | |
|---|---|---|---|---|---|---|---|---|---|---|---|
| | | | | | | w/o EV vs. EV Pre-Mi | w/o EV vs. EV GW4869 | w/o EV vs. EV Vehicle | EV Pre-MI vs. EV GW4869 | EV Pre-MI vs. EV Vehicle | EV GW4869 vs. Vehicle |
| Clev. Casp. 3 / Sarc actin | 0.48 ± 0.132 | 1.84 ± 0.553 | 1.77 ± 0.317 | 14.65 ± 1.205 | **< 0.001** | 1.000 | 1.000 | **< 0.001** | 1.000 | **< 0.001** | **< 0.001** |
| Clev. Casp 7 / Sarc actin | 0.95 ± 0.173 | 1.34 ± 0.527 | 5.46 ± 1.125 | 27.78 ± 2.268 | **< 0.001** | 1.000 | 0.212 | **< 0.001** | 0.413 | **< 0.001** | **< 0.001** |

W/o: without; EV: Extracellular Vesicles; MI: Myocardial Infarction.

## Table S6 – TLR4-NFkB axis regulates *in vitro* effects of plasma-derived EV

| Variable | | w/o EV | EV Pre-MI | EV Post-MI Vehicle | EV Post-MI GW4869 | Overall *P*-value | Pairwise Comparisons | | | | | |
|---|---|---|---|---|---|---|---|---|---|---|---|---|
| | | | | | | | w/o EV vs. EV Pre-MI | w/o EV vs. EV GW4869 | w/o EV vs. EV Vehicle | EV Pre-MI vs. EV GW4869 | EV Pre-MI vs. EV Vehicle | EV GW4869 vs. Vehicle |
| Dead / total cells (%) | TAK242 (-) | 23.8 ± 3.60 | 27.1 ± 4.96 | 77.1 ± 4.28 | 29.5 ± 5.89 | **< 0.001** | *1.000* | *1.000* | ***< 0.001*** | *1.000* | ***< 0.001*** | ***< 0.001*** |
| | TAK242 (+) | 17.6 ± 2.09 | 27.6 ± 4.88 | 33.8 ± 3.25 | 27.3 ± 7.74 | | *1.000* | *1.000* | *0.794* | *1.000* | *1.000* | *1.000* |
| | *TAK242 (+) vs (-)* | *1.000* | *1.000* | ***< 0.001*** | *1.000* | | *N.A.* | *N.A.* | *N.A.* | *N.A.* | *N.A.* | *N.A.* |
| Nuclear / Cytosolic NKkB (IF) | TAK242 (-) | 0.53 ± 0.103 | 0.61 ± 0.076 | 1.19 ± 0.057 | 0.58 ± 0.114 | **< 0.001** | *1.000* | *1.000* | ***< 0.001*** | *1.000* | ***0.001*** | ***< 0.001*** |
| | TAK242 (+) | 0.62 ± 0.080 | 0.82 ± 0.024 | 0.68 ± 0.036 | 0.49 ± 0.110 | | *1.000* | *1.000* | *1.000* | *1.000* | *1.000* | *1.000* |
| | *TAK242 (+) vs (-)* | *1.000* | *1.000* | ***0.020*** | *1.000* | | *N.A.* | *N.A.* | *N.A.* | *N.A.* | *N.A.* | *N.A.* |
| Nuclear / Cytosolic NKkB (FC-WB) | TAK242 (-) | N.A. | 1.6 ± 0.61 | 4.6 ± 1.08 | 0.4 ± 0.13 | **< 0.001** | *N.A.* | *N.A.* | *N.A.* | *1.000* | ***0.004*** | ***< 0.001*** |
| | TAK242 (+) | N.A. | 0.1 ± 0.05 | 0.10 ± 0.03 | 0.4 ± 0.04 | | *N.A.* | *N.A.* | *N.A.* | *1.000* | *1.000* | *1.000* |
| | *TAK242 (+) vs (-)* | *N.A.* | *0.995* | ***<0.001*** | *1.000* | | *N.A.* | *N.A.* | *N.A.* | *N.A.* | *N.A.* | *N.A.* |

w/o: without; EV: Extracellular Vesicles; IF: immune fluorescence; MI: Myocardial Infarction; WB: western blot; FC: fold change;.

## Table S7 – Bone marrow derived macrophages cells and EV characterization

| Variable | MΦ | M1 | M2 | Overall *P*-value | Pairwise Comparisons | | |
|---|---|---|---|---|---|---|---|
| | | | | | MΦ vs. M1 | MΦ vs. M2 | M1 vs. M2 |
| INOS ($2^{-\Delta\Delta CT}$ vs. MΦ) | 1.00 | 139.2 ± 10.23 | 1.5 ± 0.34 | **< 0.001** | **< 0.001** | 1.000 | **< 0.001** |
| TNFα ($2^{-\Delta\Delta CT}$ vs. MΦ) | 1.00 | 2.8 ± 0.68 | 0.9 ± 0.17 | **0.003** | **0.008** | 1.000 | **0.007** |
| TLR4 ($2^{-\Delta\Delta CT}$ vs. MΦ) | 1.00 | 8.7 ± 0.81 | 0.5 ± 0.05 | **< 0.001** | **< 0.001** | 1.000 | **< 0.001** |
| Arginase 1 ($2^{-\Delta\Delta CT}$ vs. MΦ) | 1.00 | 0.6 ± 0.25 | 8.6 ± 1.92 | **< 0.001** | 1.000 | **0.001** | **0.001** |
| CD206 ($2^{-\Delta\Delta CT}$ vs. MΦ) | 1.00 | 0.1 ± 0.01 | 2.7 ± 0.32 | **< 0.001** | **0.004** | **< 0.001** | **< 0.001** |
| iNOS/GAPDH (WB) | 0.3 ± 0.08 | 1.2 ± 0.12 | 0.1 ± 0.05 | **< 0.001** | **< 0.001** | 0.895 | **< 0.001** |
| TLR4/GAPDH (WB) | 9.6 ± 2.79 | 54.7 ± 6.19 | 9.3 ± 1.51 | **< 0.001** | **< 0.001** | 1.000 | **< 0.001** |
| CD68/GAPDH (WB) | 3.6 ± 1.14 | 10.2 ± 1.72 | 4.8 ± 0.94 | **0.009** | **0.012** | 1.000 | **0.039** |

WB: western blot.

## Table S8 – TLR4-NFkB axis regulates *in vitro* effects of macrophages derived EV

| Variable | | w/o EV | EV-MΦ | EV-M1 | EV-M2 | Overall *P*-value | Pairwise Comparisons | | | | | |
|---|---|---|---|---|---|---|---|---|---|---|---|---|
| | | | | | | | w/o EV vs. EV MΦ | w/o EV vs. EV M2 | w/o EV vs. EV M1 | EV MΦ vs. EV M2 | EV MΦ vs. EV M1 | EV M2 vs. EV M1 |
| Dead / total cells (%) | TAK242 (-) | 17.6 ± 1.92 | 21.3 ± 2.85 | 41.3 ± 2.09 | 11.8 ± 2.06 | **< 0.001** | *1.000* | *1.000* | ***< 0.001*** | *0.112* | ***< 0.001*** | ***< 0.001*** |
| | TAK242 (+) | 16.7 ± 1.94 | 17.1 ± 2.23 | 20.33 ± 2.24 | 15.2 ± 2.04 | | *1.000* | *1.000* | *1.000* | *1.000* | *1.000* | *1.000* |
| | *TAK242 (+) vs (-)* | *1.000* | *1.000* | ***< 0.001*** | *1.000* | | *N.A.* | *N.A.* | *N.A.* | *N.A.* | *N.A.* | *N.A.* |
| Nuclear / Cytosolic NF kB (IF) | TAK242 (-) | 0.52 ± 0.059 | 0.78 ± 0.038 | 1.05 ± 0.035 | 0.57 ± 0.024 | **< 0.001** | ***0.007*** | *1.000* | ***< 0.001*** | *0.080* | ***0.003*** | ***< 0.001*** |
| | TAK242 (+) | 0.63 ± 0.063 | 0.76 ± 0.026 | 0.69 ± 0.039 | 0.66 ± 0.080 | | *1.000* | *1.000* | *1.000* | *1.000* | *1.000* | *1.000* |
| | *TAK242 (+) vs (-)* | *1.000* | *1.000* | ***< 0.001*** | *1.000* | | *N.A.* | *N.A.* | *N.A.* | *N.A.* | *N.A.* | *N.A.* |
| Nuclear / Cytosolic NFkB (FC-WB) | TAK242 (-) | N.A. | 0.9 ± 0.18 | 2.19 ± 0.37 | 0.4 ± 0.13 | **0.001** | *N.A.* | *N.A.* | *N.A.* | *1.000* | ***0.018*** | ***0.001*** |
| | TAK242 (+) | N.A. | 0.8 ± 0.14 | 0.8 ± 0.21 | 0.4 ± 0.20 | | *N.A.* | *N.A.* | *N.A.* | *1.000* | *1.000* | *1.000* |
| | *TAK242 (+) vs (-)* | *N.A.* | *1.000* | ***0.010*** | *1.000* | | *N.A.* | *N.A.* | *N.A.* | *N.A.* | *N.A.* | *N.A.* |

EV: Extracellular Vesicles; IF: immune fluorescence; MI: Myocardial Infarction; FC: fold change; WB: western blot.

## Table S9 – EV mRNA content and concentration after MI

| NTA – Cumulative concentration (AUC) | EV Pre-MI | EV Post-MI Vehicle | EV Post-MI GW4869 | Overall *P*-value | Pairwise Comparisons | | |
|---|---|---|---|---|---|---|---|
| | | | | | Pre-MI vs. Vehicle | Pre-MI vs. GW4869 | Vehicle vs. GW4869 |
| Plasma derived EV 24h post-MI | 0.19 ± 0.038 | 0.61 ± 0.095 | 0.26 ± 0.039 | **< 0.001** | **< 0.001** | 1.000 | **0.002** |
| Plasma derived EV 48h post-MI | 0.19 ± 0.038 | 0.13 ± 0.037 | 0.21 ± 0.044 | **1.000** | N.A. | N.A. | N.A. |

| mRNA EV content | EV Pre-MI | EV Post-MI Vehicle | EV Post-MI GW4869 | Overall *P*-value |
|---|---|---|---|---|
| iNOS | n.d. | n.d. | n.d. | N.A. |
| INFγ | n.d. | n.d. | n.d. | N.A. |
| IL1α | n.d. | n.d. | n.d. | N.A. |
| IL1β | 0.99 ± 0.711 | 1.12 ± 0.956 | 0.77 ± 0.627 | 0.951 |
| RANTES | 1.15 ± 0.116 | 1.22 ± 0.395 | 1.38 ± 0.213 | 0.825 |
| IL6 | 1.50 ± 0.981 | 2.17 ± 0.315 | 2.62 ± 0.029 | 0.523 |

EV: Extracellular Vesicles; MI: Myocardial Infarction; AUC: area under the curve.

## Table S10 – *In-vitro* cleaved caspase 3 and 7 activation.

| Variable | w/o EV | EV Pre-MI | EV Post-MI GW4869 | EV Post-MI Vehicle | Overall *P*-value | Pairwise Comparisons | | | | | |
|---|---|---|---|---|---|---|---|---|---|---|---|
| | | | | | | w/o EV vs. EV Pre-Mi | w/o EV vs. EV GW4869 | w/o EV vs. EV Vehicle | EV Pre-MI vs. EV GW4869 | EV Pre-MI vs. EV Vehicle | EV GW4869 vs. Vehicle |
| Clev. Casp. 3 / Sarc actin | 4.61 ± 0.534 | 5.89 ± 0.500 | 6.14 ± 0.687 | 12.66 ± 1.122 | **< 0.001** | 1.000 | 1.000 | **< 0.001** | 1.000 | **< 0.001** | **< 0.001** |
| Clev. Casp 7 / Sarc actin | 3.17 ± 0.476 | 2.45 ± 0.216 | 3.56 ± 0.287 | 8.07 ± 1.159 | **< 0.001** | 1.000 | 1.000 | **< 0.001** | 1.000 | **< 0.001** | **< 0.001** |

W/o: without; EV: Extracellular Vesicles; MI: Myocardial Infarction.

## Table S11 – NFkB nuclear translocation in isolated adult cardiomyocytes.

| Variable | EV Pre-MI | EV Post-MI Vehicle | EV Post-MI GW4869 | Overall *P*-value | Pairwise Comparisons | | |
|---|---|---|---|---|---|---|---|
| | | | | | Pre-MI vs. Vehicle | Pre-MI vs. GW4869 | Vehicle vs. GW4869 |
| Nuclear NFkB / cytoplasmic NFkB | 0.023 ± 0.0021 | 0.048 ± 0.0052 | 0.028 ± 0.0014 | **< 0.001** | **< 0.001** | 1.000 | **0.002** |

EV: Extracellular Vesicles; MI: Myocardial Infarction.

## Table S12 – *In-vitro* macrophages derived EV cytotoxic effect under hypoxia.

| Variable | w/o EV | EV-MΦ | EV-M1 | EV-M2 | Overall *P*-value | Pairwise Comparisons | | | | | |
|---|---|---|---|---|---|---|---|---|---|---|---|
| | | | | | | w/o EV vs. EV MΦ | w/o EV vs. EV M2 | w/o EV vs. EV M1 | EV MΦ vs. EV M2 | EV MΦ vs. EV M1 | EV M2 vs. EV M1 |
| Dead cells / total cells (%) | 57.3 ± 2.60 | 52.2 ± 2.84 | 81.0 ± 3.02 | 60.3 ± 4.31 | **< 0.001** | 1.000 | 1.000 | **< 0.001** | 0.581 | **< 0.001** | **0.001** |

## Table S13 – Extracellular vesicles cytotoxic effects are mediated by EV surface proteins.

| Variable | | w/o EV | EV Pre-MI | EV Post-MI Vehicle | EV Post-MI GW4869 | Overall *P*-value | Pairwise Comparisons | | | | | |
|---|---|---|---|---|---|---|---|---|---|---|---|---|
| | | | | | | | w/o EV vs. EV Pre-MI | w/o EV vs. EV GW4869 | w/o EV vs. EV Vehicle | EV Pre-MI vs. EV GW4869 | EV Pre-MI vs. EV Vehicle | EV GW4869 vs. Vehicle |
| Dead / total cells (%) | Untreated EV | 33.3 ± 5.86 | 41.4 ± 1.98 | 81.3 ± 4.76 | 48.8 ± 5.42 | **< 0.001** | *1.000* | *0.537* | ***< 0.001*** | *1.000* | ***< 0.001*** | ***0.001*** |
| | Acid-washed EV | N.A. | 40.2 ± 5.73 | 56.3 ± 3.14 | 52.2 ± 2.40 | | *N.A.* | *N.A.* | *N.A.* | *1.000* | *0.435* | *1.000* |
| | *Untreated vs. Acid-washed EV* | *N.A.* | *1.000* | ***0.010*** | *1.000* | | *N.A.* | *N.A.* | *N.A.* | *N.A.* | *N.A.* | *N.A.* |

| Variable | | w/o EV | EV-MΦ | EV-M1 | EV-M2 | Overall *P*-value | Pairwise Comparisons | | | | | |
|---|---|---|---|---|---|---|---|---|---|---|---|---|
| | | | | | | | w/o EV vs. EV MΦ | w/o EV vs. EV M2 | w/o EV vs. EV M1 | EV MΦ vs. EV M2 | EV MΦ vs. EV M1 | EV M2 vs. EV M1 |
| Dead / total cells (%) | Untreated EV | 41.4 ± 1.98 | 23.5 ± 2.09 | 61.5 ± 5.62 | 32.8 ± 5.57 | **< 0.001** | *0.219* | *1.000* | ***0.044*** | *1.000* | ***< 0.001*** | ***0.001*** |
| | Acid-washed EV | N.A. | 28.0 ± 4.32 | 30.1 ± 1.68 | 28.3 ± 3.03 | | *N.A.* | *N.A.* | *N.A.* | *1.000* | *1.000* | *1.000* |
| | *Untreated vs. Acid-washed EV* | *N.A.* | *1.000* | ***< 0.001*** | *1.000* | | *N.A.* | *N.A.* | *N.A.* | *N.A.* | *N.A.* | *N.A.* |

EV: Extracellular Vesicles; MI: Myocardial Infarction.
